# Simian Immunodeficiency Virus Infection Mediated Changes in Jejunum and Peripheral SARS-CoV-2 Receptor ACE2 and Associated Proteins or Genes in Rhesus Macaques

**DOI:** 10.3389/fimmu.2022.835686

**Published:** 2022-02-25

**Authors:** Nongthombam Boby, Xuewei Cao, Kelsey Williams, Shiva Kumar Goud Gadila, Monica N. Shroyer, Peter J. Didier, Sudesh K. Srivastav, Arpita Das, Kate Baker, Qiuying Sha, Bapi Pahar

**Affiliations:** ^1^ Division of Comparative Pathology, Tulane National Primate Research Center, Covington, LA, United States; ^2^ Department of Mathematical Sciences, Michigan Technological University, Houghton, MI, United States; ^3^ Division of Immunology, Tulane National Primate Research Center, Covington, LA, United States; ^4^ Division of Veterinary Medicine, Tulane National Primate Research Center, Covington, LA, United States; ^5^ Department of Biostatistics, Tulane University, New Orleans, LA, United States; ^6^ Division of Microbiology, Tulane National Primate Research Center, Covington, LA, United States; ^7^ Department of Microbiology and Immunology, Tulane University School of Medicine, New Orleans, LA, United States; ^8^ Department of Tropical Medicine, Tulane University School of Public Health and Tropical Medicine, New Orleans, LA, United States

**Keywords:** ACE2 regulation, AGTR2, enteroids/organoids, MIP-1, mucosal immunity, rhesus macaque, SIV/HIV, transcriptomics

## Abstract

Angiotensin converting enzyme-2 (ACE2) and associated proteins play a pivotal role in various physiological and pathological events, such as immune activation, inflammation, gut barrier maintenance, intestinal stem cell proliferation, and apoptosis. Although many of these clinical events are quite significant in SIV/HIV infection, expression profiling of these proteins has not been well reported. Considering the different pathological consequences in the gut after HIV infection, we hypothesized that the expression of ACE2 and associated proteins of the Renin-angiotensin system (RAS) could be compromised after SIV/HIV infection. We quantified the gene expression of *ACE2* as well as *AGTR1/2*, *ADAM17*, and *TMPRSS2*, and compared between SIV infected and uninfected rhesus macaques (*Macaca mulatta*; hereafter abbreviated RMs). The gene expression analysis revealed significant downregulation of *ACE2* and upregulation of *AGTR2* and inflammatory cytokine *IL-6* in the gut of infected RMs. Protein expression profiling also revealed significant upregulation of AGTR2 after infection. The expression of ACE2 in protein level was also decreased, but not significantly, after infection. To understand the entirety of the process in newly regenerated epithelial cells, a global transcriptomic study of enteroids raised from intestinal stem cells was performed. Interestingly, most of the genes associated with the RAS, such as *DPP4*, *MME*, *ANPEP*, *ACE2*, *ENPEP*, were found to be downregulated in SIV infection. *HNFA1* was found to be a key regulator of ACE2 and related protein expression. Jejunum CD4+ T cell depletion and increased IL-6 mRNA, MCP-1 and AGTR2 expression may signal inflammation, monocyte/macrophage accumulation and epithelial apoptosis in accelerating SIV pathogenesis. Overall, the findings in the study suggested a possible impact of SIV/HIV infection on expression of ACE2 and RAS-associated proteins resulting in the loss of gut homeostasis. In the context of the current COVID-19 pandemic, the outcome of SARS-CoV-2 and HIV co-infection remains uncertain and needs further investigation as the significance profile of ACE2, a viral entry receptor for SARS-CoV-2, and its expression in mRNA and protein varied in the current study. There is a concern of aggravated SARS-CoV-2 outcomes due to possible serious pathological events in the gut resulting from compromised expression of RAS- associated proteins in SIV/HIV infection.

## Introduction

The renin-angiotensin system (RAS) initiates with the conversion of Angiotensin I (Ang I) to Angiotensin II (Ang II) by Angiotensin-converting enzyme (ACE). Ang II activates Ang II receptors 1 (AGTR1) and 2 (AGTR2) to exhibit its biological functions (1, 2). The effect of Angiotensin converting enzyme 2 (ACE2) as a critical regulator in the RAS has been extensively investigated. ACE2 is a transmembrane monopeptidyl carboxypeptidase enzyme distributed in multiple tissues across the intestines, heart, reproductive organs, kidney, brain, liver, adipose tissue, and respiratory tract. ACE2 hydrolyzes Ang I and Ang II into Ang 1-9 and Ang 1-7, respectively (3, 4). The failure of ACE2 to do so allows ACE to act on the Ang I and release Ang II which, after binding with AGTR1, induces various deleterious effects including apoptosis, inflammation, vasoconstriction, hypertension, cardiac hypertrophy, collagen production, reactive oxygen species by overproduction of TGF-β, and expression of ICAM-1, VCAM-1, and MCP-1 (2). Unlike Ang II, Ang 1-9 and Ang 1-7 protect against effects of Ang II with different mechanisms after binding with AGTR2 and Mas1 receptors, respectively (5–7). This clearly indicates the important role of ACE2 in maintaining the normal physiological state by counteracting the adverse effects of Ang II.

The role of ACE2, ACE and their peptides has been well recognized in different inflammatory conditions including acute pancreatitis, lung injury, pulmonary hypertension, cardiac hypertrophy, sepsis, and glomerulonephritis (8–11). Recent reports have also demonstrated that ACE2 is highly expressed in differentiated enterocytes targeted by severe acute respiratory syndrome corona virus-2 (SARS-CoV-2) in inducing a generic viral response program including type III interferon responses (12, 13). ACE2 is highly expressed in the small intestine compared to all other tissues, based on mRNA expression levels in different human tissues (14). Unlike in other organs, ACE2 in the gut has a completely different RAS-independent function maintaining a variety of cellular processes including intestinal amino acid homeostasis, antimicrobial peptide expression, gut microbiome modulation, immune activation, inflammation, dysbiosis, gut barrier maintenance, and even intestinal stem cell proliferation and differentiation (15–18). ACE2 is necessary for the expression of neutral amino acid transporters, such as B°AT1, and regulates uptake of neutral amino acids, like tryptophan, in the intestine (19, 20). Tryptophan is well known for its function in defense mechanisms, including lymphoid pro-inflammatory cytokine downregulation, tight junction formation, release of antimicrobial peptides, and modulation of mucosal cell autophagy (17, 21). Impaired ACE2 expression in the intestine could lead to microbial dysbiosis, leaky gut, and inflammation. Loss of intestinal barrier function and subsequent translocation of luminal bacteria is now thought to be the major cause of the chronic systemic immune activation that perpetuates HIV replication and progression to AIDS (22–26). Therefore, ACE2 could be an important player in modulating intestinal homeostasis during HIV/SIV infection, however, its role in regulating mucosal barrier function and disease pathogenesis is not well understood.

Besides ACE2, the other RAS components also play a key role in regulating gut physiological events. It is believed that while AGTR1 causes harmful effects after being activated by Ang II, AGTR2 counteracts the function of AGTR1 (15). However, there is evidence that AGTR2 promotes intestinal cell apoptosis in the presence of increased Ang II expression in an *in vitro* cell culture system (27). Therefore, it is critically important to understand the expression profile of AGTR2 in HIV/SIV infection and its impact in SIV mediated gut homeostasis.

ACE2 expression could also be affected by disintegrin and metalloproteinase domain 17 (ADAM17) and Transmembrane serine protease 2 (TMPRSS2) enzymes, which cleave and process ACE2 (28). ADAM17 promoted shedding of ACE2 into the extracellular environment after SARS-CoV spike protein bound to ACE2 (29, 30). TMPRSS2, which is highly expressed in several cell types including intestinal epithelium, lung alveolar cells, and vascular endothelium, plays an indispensable role in cellular uptake of SARS-CoV after ACE2 proteolysis (28). TMPRSS2 has also been demonstrated to accelerate influenza virus infection by cleaving the hemagglutinin receptor (31).

Due to the current COVID-19 (Coronavirus Disease 2019) pandemic, research on ACE2 expression and its role in the pathogenesis of intestinal inflammatory diseases is rising at a rapid pace (32–36). In SIV/HIV mediated intestinal enteropathy, the impact of ACE2 expression or its associated proteins AGTR1, AGTR2, ADAM17, and TMPRSS2 is not well understood. Moreover, the dynamics of lung ACE2 expression and molecular changes in enteroids grown from infected and uninfected rhesus macaques (*Macaca mulatta*; hereafter abbreviated RMs) have not been well described. The SIV infected RM model is a well-accepted model for the study of HIV-associated enteropathy and pathogenesis. Recent study also suggest that RM can be used as a preferred study species that can represent mild to moderate form of COVID-19 as observed in majority of human population (37). Bulk RNA-seq analysis of heart, lung, liver and kidney tissues from human and RM tissues also showed high level of inter-species conservation in the expression of SARS-CoV-2 and coronavirus-associated receptors and factors (SCARFs), which also suggested that RM can be used as an animal model to study COVID-19 pathogenesis (38). Hence, understanding the relative expression of these proteins and genes in HIV infected patients will be extremely valuable to evaluate its impact on gut pathogenesis as well as the mucosal CD4+ T cell population. In the present study, we used a RM model to determine the expression profiles of ACE2 and its associated proteins in the jejunum and/or lung of SIV infected RMs. We also analyzed expression of important inflammatory cytokines to understand the impact of inflammation on ACE2 expression. A global transcriptomic study on enteroids from SIV infected and uninfected jejunum crypts was performed to assess any difference in the expression of these proteins in newly regenerated cells. In the present scenario of the COVID-19 pandemic, this study will provide insight into the risk of SARS-CoV-2 infection among HIV patients.

## Materials and Methods

### Animals, Inoculation, and Tissues Collection

The study was performed using 22 Indian RMs of both sexes between 2.5 and 12.3 years of age (**Table 1**). All animals were socially housed at the Biosafety level 2 facility in the Tulane National Primate Research Center (TNPRC) in accordance with the standards incorporated in the Guide for the Care and Use of Laboratory Animals (36). All subjects were negative for HIV-2, SIV, type-D retrovirus, and simian T-cell leukemia virus type 1 infection at the beginning of this study. The Tulane Institutional Animal Care and Use Committee (IACUC) approved all animal procedures related to this study. The TNPRC is fully accredited by the Association for the Assessment and Accreditation of Laboratory Animal Care (Animal Welfare Assurance A-4499-01).

**Table 1:** List of Indian rhesus macaques examined.

Disease Category	Animal Number	Age (Year)	Sex^a^	Virus	Dosage (TCID_50_)	Route^b^	Terminal Plasma Viral Load (RNA copies/ml)	Tissue tested
Normal, uninfected	FF25	3.2	F	–	–	–	–	Lung
	GJ06	4.9	F	–	–	–	–	Lung
	FF15	6.9	F	–	–	–	–	Lung
	DJ78	8.1	F	–	–	–	–	Lung
	AG71	11.1	F	–	–	–	–	Lung
	IK15	8.5	M	–	–	–	–	Lung
Acute SIV	GI28	5.9	F	SIV_MAC251_	500	IVAG	5830000	Lung
	FT35	6.7	F	SIV_MAC251_	500	IVAG	3540000	Lung
	EK98	8.7	F	SIV_MAC251_	500	IVAG	26800000	Lung
	EM64	8.9	F	SIV_MAC251_	500	IVAG	3840000	Lung
	CF65	12.3	F	SIV_MAC251_	500	IVAG	10100000	Lung
	HV53	2.5	M	SIV_MAC251_	100	IV	340000	Lung
Pre, SIV infected	KP54	6.3	F	SIV_MAC251_	100	IV	3060000	Blood, Lung, Jejunum
	KM05	6.4	F	SIV_MAC251_	100	IV	108000	Blood, Lung, Jejunum
	KA42	7.3	F	SIV_MAC251_	100	IV	23800000	Blood
	KA76	7.4	F	SIV_MAC251_	100	IV	39100000	Blood, Lung, Jejunum
	KP60	6.4	M	SIV_MAC251_	100	IV	28400000	Blood, Lung, Jejunum
	KH79	7.2	M	SIV_MAC251_	100	IV	138000000	Blood, Jejunum
	KA78	7.3	M	SIV_MAC251_	100	IV	5510000	Blood, Jejunum
	KE75	7.3	M	SIV_MAC251_	100	IV	2280000	Blood
	JV97	7.4	M	SIV_MAC251_	100	IV	190000	Blood, Lung, Jejunum
	JK56	8.3	M	SIV_MAC251_	100	IV	6480000	Blood, Lung, Jejunum

a F and M denote female and male, respectively.

b IV and IVAG denote intravenous and intravaginal route, respectively.

TCID_50_ represents tissue culture infectivity dose at 50%.

Blood and jejunum collected from 10 RMs were studied longitudinally for several cellular and molecular assays. Lung tissue collected from three cohorts of RMs (uninfected, acutely infected, and chronically infected; 6 per group) was used for measuring ACE2 expression (**Table 1**). All acutely and chronically infected subjects were inoculated with 100 or 500 TCID_50_ pathogenic SIV_MAC_251 using either intravenous (IV) or intravaginal (IVAG) routes (**Table 1**) to mimic the major routes of HIV transmission in humans. For analysis of plasma/serum, samples were collected from various time points, including pre infection, 14-, 21-, 40-, 60-, 90-, 112-, 145-, and 180-day post infection (dpi). On the other hand, for tissues collected by resection surgery or necropsy, only three time points were used, namely pre infection, acute infection (21 dpi) and chronic infection (180 dpi). In addition, both pre infection and at 180 dpi, jejunum crypts were isolated from animals, enteroids were grown from crypts, and RNA-seq was performed for transcriptomics analysis. Small pieces of freshly collected jejunum tissues were preserved in Buffer RLT (Qiagen, Germany) and used for real-time PCR for relative quantification of differential gene expression. Freshly collected jejunum and lung tissues were fixed in zinc formalin (Anatech, Ltd., USA), processed for paraffin embedding, and used for immunohistochemistry (IHC) assays. Viral inoculation and sample collection were performed under the direction of veterinarians. All analyses except those in the lung were presented based on the longitudinal study. Due to infeasibility of sample collection, lung tissues representing each cohort of infection were obtained from different animals, and thus could not be used for the longitudinal study. In our earlier studies we did not detect any association between viral dosage, CD4 depletion, and viral loads in RMs (25, 39–41).

Every effort was made to avoid discomfort and pain to animals. At the TNPRC, animal care staff and veterinarians observed animals several times daily for signs of pain, distress, and disease, and animal discomfort and pain were alleviated by appropriate use of anesthetics and analgesics. Subjects were anesthetized intramuscularly (IM) with ketamine hydrochloride (10 mg/kg bw) or tiletamine hydrochloride/zolazepam (Telazol^®^, Zoetis, USA) (5-8 mg/kg bw) when removed from their home cage for blood collection, physical exams, and other surgical procedures. If necessary, for clinical diagnostic procedures or if major surgery was required, isoflurane gas inhalation anesthesia was used after induction with ketamine hydrochloride. Buprenorphine hydrochloride (0.01 mg/kg IM) or sustained release Buprenorphine hydrochloride (0.2 mg/kg subcutaneously) was used for post-procedural analgesia. At the end of the study, subjects were humanely euthanized using methods consistent with recommendations of the American Veterinary Medical Association (AVMA) Panel on Euthanasia.

### Quantification of Plasma Viral Load

Plasma viral RNA was measured by quantitative reverse transcription-PCR (qRT-PCR) at the Wisconsin National Primate Research Center with a lower detection limit of 60 SIV RNA copies/mL of plasma (25).

### Isolation of Lamina Propria Lymphocytes (LPL) From Jejunum

The jejunum LPL was isolated by collagenase treatment followed by Percoll (Sigma-Aldrich, USA) density gradient centrifugation as described earlier (25, 39, 42). Briefly, a jejunum section of 2-4 cm length was collected, washed with chilled sterile PBS, and minced into small pieces. The minced tissue was treated with 1 mM EDTA in HBSS and shaken for 30 min at 300 rpm, 37°C. The epithelial cells were removed by filtration through a screen cup strainer with mesh size 50 (0.229 mm, Sigma-Aldrich). The tissues on the strainer were scraped off, minced, and further digested with type II collagenase (60 U/mL) (Sigma-Aldrich). After washing, the cells were passed through a 16-gauge feeding needle for better separation of any clumps. The larger clumps were filtered out using a nylon biopsy bag (Fisher Scientific, USA). The isolated LPL was enriched by centrifugation over the prepared 60% and 35% isotonic Percoll layers at 1900 rpm for 30 min at 4°C. The enriched LPL was collected, washed, and resuspended with complete RPMI-1640 media with 10% FBS, then used for flow cytometry staining. These enriched LPLs may contain trace amounts of epithelial and other leukocyte positive cells as reported in our earlier study (43).

### Flow Cytometry

The frequency of CD4+ and CD8+ T cell populations were quantified in jejunum LPL using flow cytometry staining and analysis as done previously (39, 44). Briefly, one million isolated LPL were first stained with Live/Dead fixable aqua dead cell stain (1:100 dilution, Thermo Fisher Scientific, USA) at 37°C for 10 min. This was followed by surface staining using fluorochrome conjugated anti-CD3, anti-CD4 and anti-CD8 monoclonal antibodies (**Supplementary Table 1**), then incubation at room temperature (RT) for 25 min. After staining, cells were washed and stored at 4°C in BD stabilizing and fixative buffer. At least 50,000 events were acquired from each sample, and analyzed with FlowJo software (version 10.7.2., FLowJo LLC, USA) the next day. Only singlets and live cell populations were considered for sequential identification and frequency determination of different T cell populations.

### Immunofluorescence (IF) Staining in Jejunum

Jejunum tissue sections were processed for IF staining as described earlier (39, 41, 44). Tissue sections of 5 μM thickness were stained by incubating for 1h with rabbit polyclonal anti-ACE2 antibodies (Sino Biologicals, USA), then washed and stained for 40 min with Alexa Flour 568-conjugated secondary antibodies (Life Technologies, USA) (**Supplementary Table 1**). Negative control slides were incorporated in each experiment either by omitting the primary antibody or using isotype IgG (H+L) controls (39, 43, 45). The nuclear staining was performed with diluted DAPI (Millipore Sigma, USA) and incubated for 10 min at RT (**Supplementary Table 1**). The stained tissue sections were mounted with the Prolong Gold antifade reagent (Invitrogen, USA). For cytokeratin and ACE2 dual staining, tissues were first stained with rabbit anti-cow cytokeratin wide spectrum polyclonal antibody (Dako, USA) diluted in serum-free blocking buffer, then incubated at RT for 1h as we reported earlier (45). After washing twice in TBS buffer, the slides were incubated with the MACH3 rabbit probe (BioCare Medical, USA) followed by MACH3 AP-polymer (BioCare Medical) for 20 min each. The sections were monitored under a microscope and allowed to develop color using permanent red chromogen (Dako) at 1:100 dilution. To visualize the coexpression of ACE2 and cytokeratin proteins, the tissue sections were further stained sequentially with ACE2 antibodies, followed by Alexa 488 conjugated anti-Rabbit secondary antibodies (Invitrogen), and finally with DAPI as described above.

Imaging was performed with a Ti2-E motorized fluorescence microscope (Nikon, USA) using a 20x objective, with a resolution of 2048 × 2044 pixels. Control and experimental slides were imaged during the same session with identical acquisition parameters. Fluorescence intensity was optimized on isotype control tissues to eliminate tissue autofluorescence and remained constant for all the experimental slides. To quantify the mean fluorescence intensity (MFI), regions of interests (ROI) were manually drawn (**Supplementary Figure 1A**) on the epithelial regions of 20-23 randomly selected villi. Nikon NIS Elements software was used to measure MFI.

### IHC Staining in Jejunum and Lung

IHC assays were performed to quantify the expression of different proteins of interest in jejunum and lung tissues (**Table 1**) using the Mach3 Rabbit AP-polymer Detection Kit (Biocare Medical) as described previously (45). Five μM paraffin-embedded tissue sections were deparaffinized by being placed at 60°C overnight and sequentially treated with xylene and ethanol. Epitope retrieval was achieved by heating tissues stored in a citrate buffer (Vector Laboratories, USA). After blocking with a serum-free protein blocker (Vector laboratories), the tissues were incubated for 1h at RT with antibodies against either anti-ACE2, anti-AGTR2, or anti-TMPRSS2 proteins (**Supplementary Table 1**). Since lung tissue usually produces strong autofluorescence, expression of ACE2 in the lung was quantified by IHC. A negative control sample treated with rabbit IgG fractions was also included in every experiment. The tissues were incubated with the kit’s probe and polymer as directed, and finally developed using permanent red chromogen for lung ACE2 staining and BCIP/NBT chromogen system (Abcam, USA) for the detection of AGTR2 and TMPRSS2 proteins in jejunum. Slides were mounted using Vecta Mount AQ (Vector Laboratories).

The whole stained tissue were scanned at 20x objective using Axio slide scanner (Zeiss, Germany). For quantification of AGTR2+ cell density (counts/mm^2^), 20 equal size ROIs were manually drawn in the lamina propria region and analyzed with multiplex IHC module (v3.0.4) of the Halo software (Indica Labs, USA). For lung ACE2, ROIs were manually drawn on the epithelium of every bronchiole in the tissue section, and the area of ACE2 positive tissue was calculated per total ROI and expressed as a percentage using Indica Labs’ area quantification module (v2.1.11) as described earlier (46). TMPRSS2 expression in the villi epithelium was quantified by gating ROI in the epithelium of 20 randomly selected villi using a similar analysis module as with the lung ACE2 (**Supplementary Figures 1B, C**). As the crypt epithelium also showed TMPRSS2 expression, we performed a separate analysis of TMPRSS2 expression in this region by selecting 30 random crypts.

### Isolation of Jejunum Crypts and Generation of Enteroid Culture

Jejunal crypts were isolated using the low-temperature method with modification, as described previously (26, 47). Briefly, a piece of jejunum (3-5 cm) was collected and thoroughly cleaned with sterile PBS. The tissue was minced into small pieces, treated with 5 mM EDTA (Thermo Fisher Scientific) prepared with 1mM Dithiothreitol (DTT, Thermo Fisher Scientific) in 1x HBSS, kept on ice, and constantly stirred at 200 rpm for 5 min. The undigested tissue pieces were allowed to settle down and the supernatant was discarded. The undigested tissues were treated with a pH 7.3 chelating buffer containing 27 mM Na_3_C_6_H_5_O_7_ (G-Biosciences, USA), 5 mM Na_2_HPO_4_ (USB corporation, USA), 96 mM NaCl (Sigma-Aldrich), 8 mM KH_2_PO_4_ (VWR, USA), 1.5 mM KCl (Thermo Fisher Scientific), 0.5 mM DTT, 55 mM D-sorbitol (VWR), and 44 mM sucrose (VWR). Tissues were treated alternatively with EDTA and chelating buffer for a total of four times. The crypts were isolated by tapping or shaking vigorously in the fresh chelating buffer, then filtered through a 100 μM cell strainer, suspended with 1 volume of DMEM (Thermo Fisher Scientific) supplemented with 1% BSA (Sigma-Aldrich), and centrifuged at 4°C and 200 g for 10 min. The isolated crypts were resuspended in DMEM with 1% BSA, and the number of viable crypts was counted.

Enteroids were grown from the isolated crypts following the protocol for human intestinal stem cells (ISC) with modification (26, 48, 49). Briefly, crypts resuspended in DMEM with 1% BSA were mixed with an equal volume of BD Matrigel basement membrane to make a cell concentration of 1000 crypts/50 μL. 50 μl of the crypt suspension was loaded onto each well of a pre-warmed 24-well cell culture plate (Corning, USA) and kept at 37°C for 10 min. After the gel was solidified, 750 μL of pre-warmed complete seeding media was added to each well, then incubated at 37°C in a 5% CO_2_ for a total duration of 13 days. The complete seeding medium contained 2 mM glutamine (Life Technologies), 10 mM HEPES (Life Technologies), 100 U/mL penicillin (Life Technologies), 100 μg/mL streptomycin (Life Technologies), 1x N2 supplement (Life Technologies), 1x B27 supplement (Life Technologies), 1% BSA (Sigma-Aldrich), 50% WNT-3A-conditioned medium prepared in-house using L WNT-3A cell line (ATCC, USA), 1 μg/mL R-Spondin 1 (R&D Systems, USA), 1 mM N-acetylcysteine (Sigma-Aldrich), 500nM A-83-01 (Peprotech, USA), 10 nM [Leu]15-Gastrin (Sigma-Aldrich), 10 mM Nicotinamide (Peprotech), 50 ng/mL EGF (Sigma-Aldrich), 100 ng/mL Noggin 1 (R&D Systems), and 10 μM SB202190 (Sigma-Aldrich) in an advanced DMEM/F12 medium (Life Technologies). During the first two days of culture, the culture medium was supplemented with 2.5 μM thiazovivin (Stemgent, USA) and 2.5 μM CHIR99021 (Stemgent). The seeding medium was replaced with 750 μL of fresh pre-warmed complete medium every 2 days. Fully grown enteroids were harvested using a gentle cell dissociation agent (Stemcell Technologies Inc., USA), usually on 13^th^ day of culture. The dissociated cells were spun down at 290 x g for 5 min at 4°C after 2-3 washes with DMEM (1% BSA). The cell pellet was resuspended in Buffer RLT and stored at –80°C until used for RNA isolation.

### RNA Isolation, cDNA Preparation and Real-Time PCR

The frozen jejunum tissues stored in Buffer RLT were thawed at RT, minced, and vortexed at high speed for 1-2 min. The lysate was homogenized by spinning at 11,000 rpm for 2 min using a QIAshredder spin-column (Qiagen). The supernatant was collected and mixed with an equal volume of 70% ethanol. The lysate was subsequently used for RNA isolation using the RNeasy mini kit (Qiagen) following the manufacturer’s instruction. The quality and quantity of the isolated total RNA were assessed in a Bioanalyzer 2100 system using the RNA 6000 Pico kit (Agilent Technologies, USA). cDNA from the isolated RNA was prepared following the protocol of the Superscript IV first-strand synthesis protocol (Thermo Fisher Scientific). The RNA in the final cDNA product was removed by adding 1 μL of RNase H/20 μL of reaction mixture, and incubated at 37°C for 20 min. The cDNA was then stored at -20°C until used.

qRT-PCR was performed to determine mRNA abundance of different proteins and cytokines. Relative abundance of each mRNA type was quantified using a set of gene specific primers (**Supplementary Table 2**) developed using an online primer designing tool (Integrated DNA Technologies, USA). qRT-PCR was performed using PowerUp SYBR green master mix (Applied Biosystems, USA) in a 7900-HT fast real-time PCR system (Applied Biosystems). The thermal cycling comprised a single step of 50°C for 2 min; 95°C for 2 min; 40 cycles of 95°C for 15 sec; and 60°C for 1 min. Before comparing between samples, each gene expression in every sample was normalized against that of an internal control GAPDH to account for any variations. Relative gene expression was determined from the means of change in threshold cycle (2^-ΔCt^) as described earlier (50).

### RNA Isolation From Enteroid and Generation of RNA-Seq Data

Total RNA from enteroids was isolated and quantified as mentioned previously (26). A cDNA library from the enteroid RNA was constructed at Novogene using a NEBNext^®^ Ultra RNA Library Prep Kit for Illumina^®^ (cat# E7420S, New England Biolabs, USA) following manufacturer protocol. This included enrichment of mRNA through ribosomal RNA removal, random mRNA fragmentation using divalent cation at elevated temperature, first cDNA strand synthesis using random hexamers, and second cDNA strand synthesis using dNTPs, DNA polymerase I and RNase H. Finally, the double stranded cDNA library was constructed after a series of terminal repair and ligation processes. cDNA libraries of 250-350 bp were preferentially selected and enriched with Phusion High-Fidelity DNA polymerase-based PCR. The quantity and quality of the resulting cDNA was determined by a Qubit fluorometer (Thermo Fisher Scientific) and Agilent 2100 Bioanalyzer (Agilent Technologies), respectively. The cDNA libraries were finally sequenced on an Illumina Nova Seq 6000 platform (Illumina, USA). Forty million raw reads were generated from each library and stored in the FASTQ format using (bcl2fastq2) conversion software (v2.17).

### Transcriptome Assembly

The quality of raw reads in FASTQ format was checked using FastQC (v0.11.9 released: http://www.bioinformatics.babraham.ac.uk/projects/fastqc/). FastQC showed few overrepresented sequences for each library and a high per base/tile sequence quality, exceeding 34 on the Phred scale (less than 1/2000 chance of a base being wrong). The raw reads were mapped to the reference sequences and annotation of the RM (https://support.illumina.com/sequencing/sequencing_software/igenome.html) using TopHat2. Reads with multiple alignments were discarded, and gene expression counts were calculated using htseq-count in Galaxy platform (https://usegalaxy.org/).

### Differential Gene Expression Analysis

To determine differentially expressed genes (DEGs) between enteroids from infected and uninfected RMs, transcriptomic profiling and data analysis were performed using DESeq2 in R/Bioconductor software (https://bioconductor.org/packages/release/bioc/html/DESeq2.html). First, genes with read counts smaller than 10 were excluded from further analysis. We then transformed the read counts to log2 scale using regularized-logarithm transformation (rlog). Principal component analysis (PCA) was then applied to provide insight into associations between samples, and to identify subgroups (in our case, infected and uninfected) and outliers. A differentially expressed gene was identified if the gene expression in the enteroid from infection with false discovery rate (FDR) < 0.05 and the absolute value of log2 fold-change > 1 when compared with that of uninfected control. The FDR referred to the adjusted *p* value obtained by applying Benjamin and Hochberg’s (BH) correction on the original *p* value; the fold-change indicated the degree of change of gene expression. A heat map depicting only the proteins of interest and their associated proteins was generated using the “*pHeatmap*” R package (https://cran.r-project.org/web/packages/pheatmap/index.html), enabling the detection of patterns of differential gene expression in the enteroids from infected and uninfected.

### Pathway Enrichment Analysis

To better understand the involvement of any biological functions or pathways during infection, a pathway enrichment analysis was performed based on the Kyoto Encyclopedia of Genes and Genomes (KEGG) pathways. All DEGs were mapped to the KEGG pathways using a functional annotation tool named Database for Annotation, Visualization, and Integrated Discovery Bioinformatics Resource (DAVID: https://david.ncifcrf.gov/). Significantly enriched pathways were identified by DEGs if FDR < 0.05. Moreover, genes weighted by length and categories with FDR < 0.05 were identified as being significantly enriched in the corresponding pathways.

### Ingenuity Upstream Regulator Analysis

DEGs were further analyzed using QIAGEN’s Ingenuity^®^ Pathway Analysis software (IPA^®^, https://www.qiagenbioinformatics.com/products/ingenuitypathway-analysis). Its novel upstream regulator analysis (URA) tool can identify potential transcriptional regulators (49, 50), determine how many known targets or regulators are contained within the dataset and compare each differentially expressed molecule to the reported relationship in the literature. The URA tool is based on prior knowledge of expected effects between transcriptional regulators and their target genes as stored in the Ingenuity^®^ Knowledge Base. Two statistical measures, an overlap *p*-value and an activation z-score, were computed for each potential transcriptional regulator. Activation z-scores indicate the activation states of the regulators; a score >2.0 indicates that a target molecule in the dataset is activated, whereas a score of <−2.0 indicates that it is inhibited. The overlap *p*-value measures whether there is a statistically significant overlap between the dataset molecules and those regulated by an upstream regulator. It was calculated using Fisher’s Exact Test and significance is attributed to values < 0.05.

### Quantification of Soluble CD14 Marker for Monocyte Activation and Microbial Translocation

Plasma CD14 levels at pre, acute and chronic time points from 10 RMs were measured using a quantitative Human CD14 sandwich ELISA (Human CD14, Duoset ELISA, R&D Systems) in duplicates following the manufacturer’s recommendation. The detection limit of this assay was from 62.5-4000 pg/mL. Each sample was diluted 2000 fold prior to run this assay. The absorbance was recorded using the Synergy H4 microplate reader (Biotek Instrument, Inc., USA).

### ACE2 Quantification in Plasma

Measurement of total circulating ACE2 levels from frozen plasma samples was performed by ELISA using an ACE2 ELISA kit (R&D Systems) following manufacturer instruction with minor modification. Briefly, the microtiter plates were coated with goat anti-human ACE2 capture antibodies overnight. After washing, the plasma samples were added at serial two-fold dilutions and incubated at RT for 2h followed by overnight incubation at 4°C. The wells were washed, and the plates were developed by consecutive treatment with biotin-conjugated goat anti-human ACE2, streptavidin conjugated horseradish peroxidase, and TMB substrate. The reaction was then stopped, and the absorbance was recorded at 450 nm using the Synergy H4 microplate reader (Biotek Instrument, Inc.). All samples were assayed in duplicate with appropriate positive and negative controls. For quantification of total ACE2 plasma level, a standard curve with known ACE2 concentrations was generated. Nonlinear regression using a sigmoidal dose-response variable slope model was used to interpolate concentrations from the standard curve.

### Angiotensin II (Ang II) Quantification in Plasma

An Angiotensin II competitive ELISA kit (Enzo Life Sciences Inc., USA) was used to measure the concentration of plasma Ang II following manufacturer instruction. Briefly, frozen plasma samples were thawed and used in duplicate. Plasma samples were added to the respective wells coated with goat anti-rabbit IgG antibody and incubated with polyclonal anti-Ang II antibody on a shaker at RT for 1h. After washing, biotin-conjugated Ang II was added and incubated on a shaker at RT for 1h. The wells were washed, treated with streptavidin conjugated horseradish peroxidase, and shaken for 1h at RT. Finally, the plate was washed and developed with TMB substrate for 30 min. The reaction was then stopped, and the wells were read at 450 nm optical density where the intensity of signal is inversely proportional to the level of Ang II. Nonlinear regression using a sigmoidal dose-response variable slope model was used to interpolate concentrations from the known standard curve.

### Quantification of AGTR1in Plasma

AGTR1 plasma concentration was measured using an AGTR1 sandwich ELISA kit (LSBio, USA) following manufacturer instruction. The plate was developed with TMB substrate. The wells were read at 450 nm optical density where the amount of signal is inversely proportional to the level of AGTR1. Nonlinear regression using a sigmoidal dose-response variable slope model was used to interpolate concentrations from the known standard curve.

### Quantitative Determination of Serum Lactate Dehydrogenase (LDH) Activity

Serum collected from serum clot tubes was analyzed using a Beckman Coulter AU 480 analyzer. Reagents containing lactate and NAD+ were added to the sample to measure NADH production by quantifying absorbance of light at 340 nm. The rate of change of absorbance at 340 nm is directly proportional to the LDH activity in the sample.

### Quantification of Inflammatory Cytokines and Chemokine in Plasma

Inflammatory cytokines (IL-1β, IL-6, and TNF-α) and MCP-1 chemokine (monocyte chemoattractant protein-1) in plasma were quantified using a U-plex biomarker NHP multiplex assay (Meso Scale Diagnostics, USA) following manufacturer instruction with minor modification. Firstly, the wells in the U-plex plate were coated with biotinylated capture antibody and incubated overnight at 4°C. After washing the plate, calibrator standards and samples were added to the wells and incubated overnight at 4°C. After three washes, detection antibody was added to each well and incubated on a shaker at RT for 1h. Finally, the plate was washed, read buffer was added to each well, and the plate was read immediately on an MSD microplate reader (Meso Scale Diagnostics). The concentration of each cytokine was determined based on the standard curve plotted between the known concentration of calibration standards and their respective signal.

### Statistical Analysis

All statistical analyses and graphical representations in the present study were performed using GraphPad Prism version 9 (GraphPad Software, USA). One-way ANOVA was used to observe any statistically significant differences between three or more groups. Bonferroni and Tukey-Kramer’s multiple comparison tests were applied for equal and unequal sample size, respectively, to identify statistically significant differences between the groups. A student T-test was applied to examine any statistical differences between two groups. Correlation analysis between different parameters was performed with a two-tailed Spearman’s correlation method. A *p*-value of < 0.05 was considered statistically significant in all analyses.

## Results

### Plasma Viral Loads in Infected RMs

All the infected subjects had detectable plasma viral loads. During acute infection the plasma viral load ranged from 3.4 X 10^5^ to 3.4 X 10^7^ copies of RNA/mL of plasma, with a mean of 1 X 10^7^ copies (n=16). The plasma viral load increased during chronic infection, ranging from 1 X 10^5^ to 1.4 X 10^8^ copies of RNA/mL of plasma, with a mean of 2.5 X 10^7^ copies (n=10). However, the difference in mean plasma viral load between acutely and chronically infected RMs was not statistically significant (*p* = 0.193).

### Dynamics of Gene Expression of Jejunal *ACE2*, *TMPRSS2*, *ADAM17*, *AGTR1* and *AGTR2* After Infection

**Figure 1:**
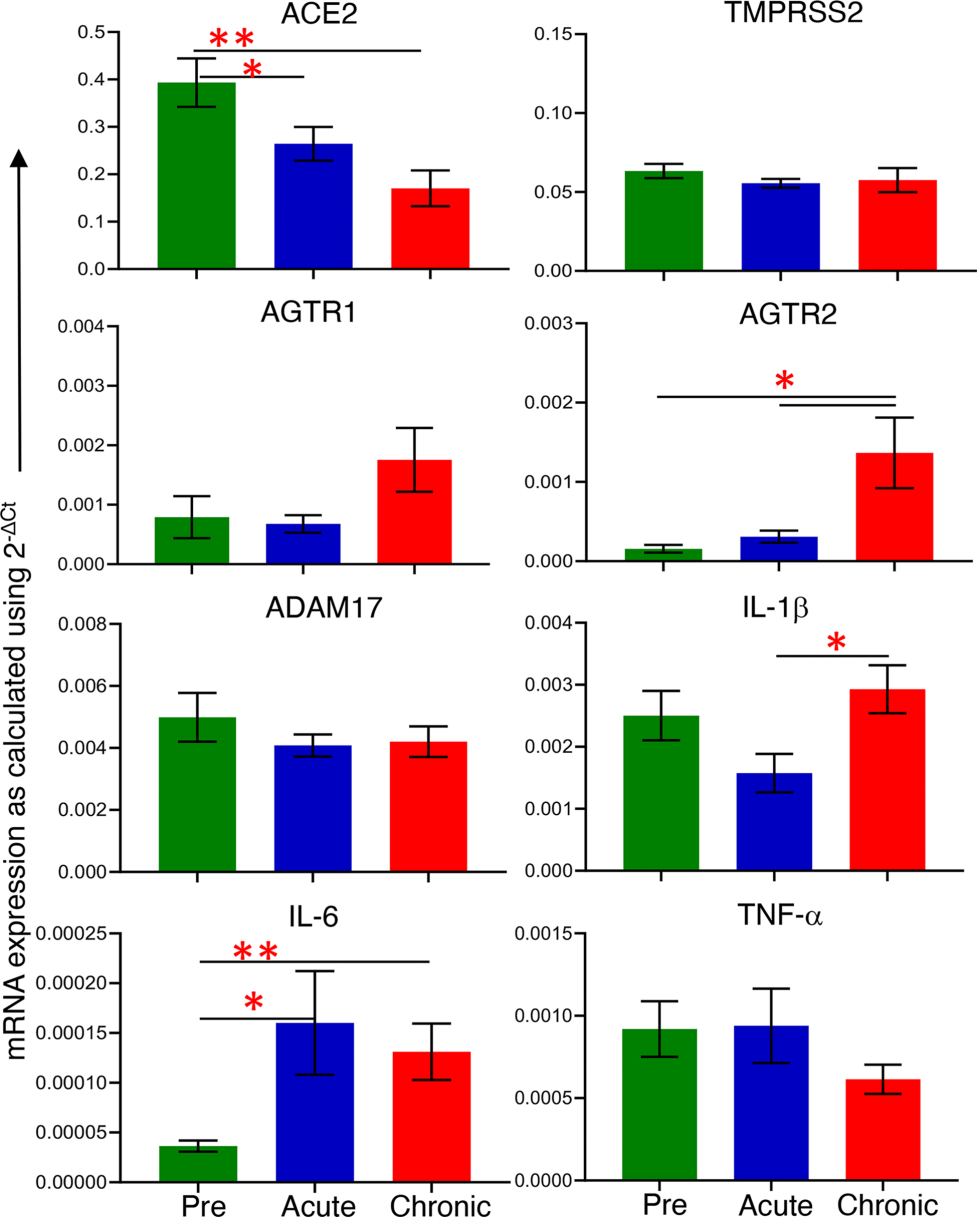
mRNA expression of *ACE2* associated proteins and inflammatory cytokines during pre and post infection. mRNA expression of protein of interest (*ACE2*, *TMPRSS2*, *AGTR1*, *AGTR2*, and *ADAM17*) and inflammatory cytokines (*IL-1β*, *IL-6*, and *TNF-α)* in whole jejunum tissue were quantified. mRNA expression for each gene was quantified relative to the internal control *GAPDH* (n=6-8). The error bars represent the mean of relative fold-change for each group ± SE. *p < 0.05, **p < 0.01 as determined by the unpaired T-test. The green, blue, and red bar graphs represent pre, acute and chronic infection time points, respectively.

Differential expression of *ACE2* as well as associated genes were examined at their transcriptional level by qRT-PCR both pre and post infection. The basal mRNA levels of these proteins were first compared with an internal control *GAPDH* to understand their expression levels in jejunum tissue. The differences between Ct of each gene and *GAPDH* were calculated, and expression was considered high if ΔCt < 5 cycles, moderate if 5 < ΔCt < 15 cycles, and low if ΔCt > 15 cycles (51). The expression of *ACE2* (mean ± SE: 1.4 ± 0.2 cycles) and *TMPRSS2* transcripts (4 ± 0.1 cycles) were identified as high, while *ADAM17* (8.1 ± 0.6 cycles), *AGTR1* (11.8 ± 0.9 cycles) and *AGTR2* transcripts (14.4 ± 1.2 cycles) were moderately expressed in the jejunum (**Supplementary Figure 2**). Next, we analyzed the relative mRNA expression of each gene and compared between pre and post infection. Notably, compared with pre infection (0.39 ± 0.05), expression of *ACE2* mRNA transcripts was significantly downregulated during acute infection (0.26 ± 0.04, *p* = 0.04) and chronic infection (0.17 ± 0.04, *p* = 0.002) by 1.5 and 2.3-fold, respectively (**Figure 1**). There was no statistically significant difference in *TMPRSS2*, *AGTR1*, and *ADAM1*7 mRNA expression after infection (**Figure 1**). In contrast, compared to pre infection (0.00016 ± 0.00005, *p* = 0.002) and acute infection (0.0003 ± 0.00008, *p* = 0.01), *AGTR2* transcripts were significantly increased during chronic infection (0.0014 ± 0.0004) by 8.8 and 4.7-fold, respectively (**Figure 1**). We did not observe any significant differences in *ACE2* mRNA expression between male and female RMs (**Supplementary Figure 3**).

### Increased Inflammatory IL-6 Negatively Correlates With *ACE2* Gene Expression

**Figure 2:**
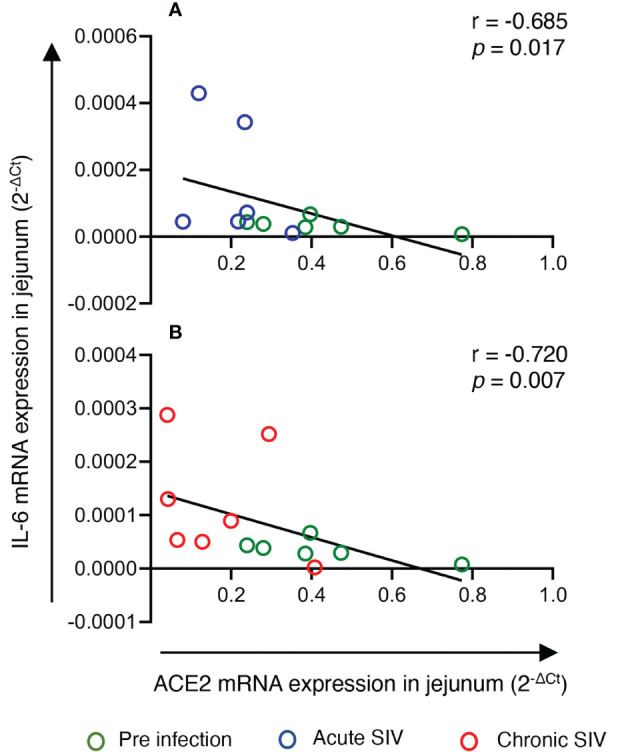
*IL-6* mRNA expression negatively correlated with jejunum *ACE2* expression during infection. mRNA expression of *ACE2* and *IL-*6 in the jejunum were measured by qRT-PCR, and correlations between these two genes at different infection stages were analyzed. **(A)** Spearman’s rank correlation analysis between *ACE* and *IL-*6 during pre (green circles) and acute (blue circles) infection showed a significant negative correlation (r = -0.685, *p* = 0.017) with the increase in *IL-6* and the reduction of *ACE2* expression (n=6). **(B)** Similarly, a significant negative correlation was detected between *IL-6* and *ACE2* mRNA expression during pre infection (green circles) and chronic infection (red circles) (r= -0.720, *p* = 0.007) (n=6-8).

To determine whether the changes in *ACE2* and *AGTR2* gene expression detected in jejunum during infection were also linked with mucosal inflammatory cytokines, we quantified mRNA expression of three important inflammatory cytokines (*IL-1β*, *IL-6*, and *TNF-α*) from total RNA isolated from jejunum (n=6-8) by qRT-PCR. We first determined the expression of each gene in the jejunum tissue by calculating ΔCt between the gene of interest and the internal control *GAPDH*. The calculated ΔCt ranged between 5 and 15 for all the genes (mean ± SE: *IL-1β* = 8.9 ± 0.3 cycles, *IL-6* = 15 ± 0.4, and *TNF-α* = 10.5 ± 0.5), indicating moderate expression of these genes in jejunum (**Supplementary Figure 4**). Expression analysis of *IL-1β* transcripts at different infection time points showed the lowest expression during acute infection (0.0016 ± 0.0003) where the values were nearly the same as in pre infection (0.0025 ± 0.0004). However, *IL-1β* mRNA expression was significantly increased during chronic infection (0.0029 ± 0.0004, *p* = 0.01) only compared to acute infection (**Figure 1**). Expression of *IL-6* mRNA transcripts was significantly upregulated during acute (5-fold; mean ± SE: 0.0002 ± 0.00005, *p* = 0.03) and chronic (2.5-fold; 0.0001 ± 0.00003, *p* = 0.006) compared to pre infection (0.00004 ± 0.000006) (**Figure 1**). We were unable to detect any change in *TNF-α* mRNA transcript expression level during pre (0.0009 ± 0.0002), acute (0.0009 ± 0.0002), and chronic infection (0.0006 ± 0.0001) (**Figure 1**). Since *IL-6* mRNA expression was significantly upregulated during acute and chronic infection, we performed a correlation analysis between *IL-*6 and *ACE2* as well as *IL-6* and *AGTR2* mRNA expressions. A two-tailed Spearman’s rank correlation coefficient analysis between *IL-6* and *ACE2* mRNA expressions indicated a highly significant negative correlation between the changes observed in *IL-6* and *ACE2* expression level during pre and acute infection (*p* = 0.017, **Figure 2A**) as well as during pre and chronic infection (*p*= 0.007, **Figure 2B**). However, we did not see any significant correlation between *IL-6* and *AGTR2* at any time point (data not shown).

### Decreased *DPP4*, *MME*, *ANPE*P, *ACE2*, *ENPEP*, and *SOX9* Gene Expression in Enteroids From Infected RMs

**Figure 3:**
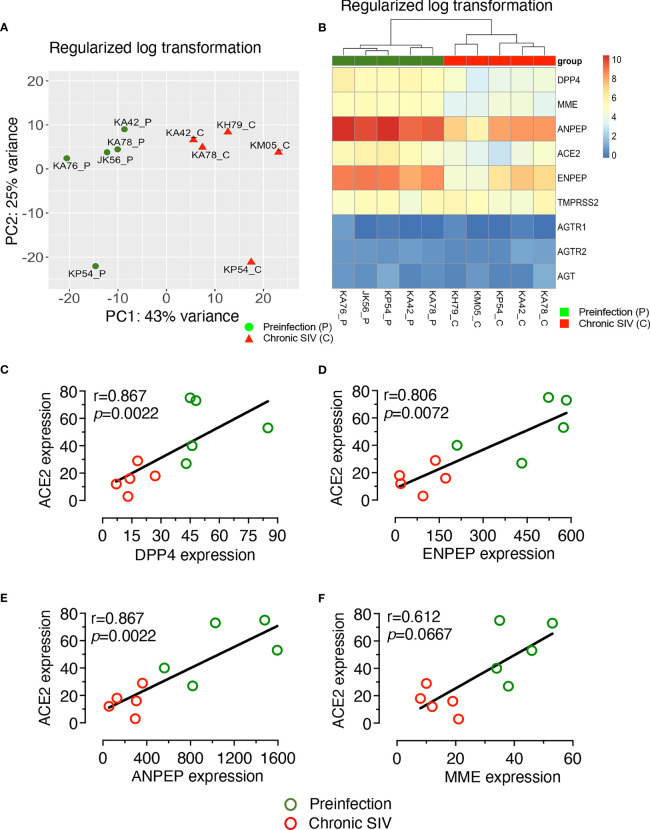
Global transcriptomic profiling of enteroids from infected and uninfected RMs. Enteroids were grown after isolating crypts from the jejunum and total RNA was isolated for the RNA-seq analysis. **(A)** PCA plot of enteroids from 5 uninfected (JK56, KA42, KA76, KA78, and KP54) and 5 chronically infected RMs (KA42, KA78, KP54, KH79, and KM05) revealed a clear separation between infected RMs and uninfected controls, along with the first principal component (PC1) with 43% of the total variance and the second principal component (PC2) with 25%. **(B)** Heatmap of differentially expressed genes (DEGs) from enteroids which encode RAS-related proteins, arranged from smallest to largest adjusted p-values. Note that there were no significant differences in *TMPRSS2*, *AGTR1*, *AGTR2*, and *AGT* gene expression between infected and uninfected enteroids. P- and C-suffix of animal numbers at the bottom of Heatmap denote pre and chronic infection time points, respectively. Spearman’s rank correlation coefficient of determination between *ACE2* and *DPP4* gene expression **(C)**, *ACE2* and *MME* gene expression **(D)**, *ACE2* and *ANPEP* gene expression **(E)**, and *ACE2* and *ENPEP* gene expression **(F)** is shown for all 5 uninfected and 5 infected macaques. Strong significantly positive correlations were detected between decreased *ACE* gene expression and reduction of *DPP4*, *ANPEP*, or *ENPEP* expression in enteroids. A positive correlation was also detected between *ACE2* and *MME* gene expression, but it was not statistically significant. **(C-F)** Green and red circles represent pre and chronic infection timepoints, respectively.

The impact of SIV infection on the expression of *ACE2*, *TMPRSS2*, *AGTR1*, and *AGTR2* genes in regenerated epithelial cells remained unclear. To determine whether the reduction of *ACE2* expression detected during chronic infection in jejunum tissues was also a failure of intestinal homeostasis and intestinal regeneration, we studied the whole transcriptomic profiles of enteroids from 5 uninfected control and 5 chronic infected. Total RNA from enteroids was isolated and transcriptomic analysis was performed to identify DEGs between these two groups. PCA analysis revealed a clear separation between infected RMs and uninfected controls along with the first principal component (PC1), with 43% of the total variance, and the second principal component (PC2), with 25% of the total variance (**Figure 3A**).

A heat map was generated showing the expression of important genes involved in the RAS, including those which were not differentially expressed (*AGTR*1, *AGTR2*, and *AGT*) in the enteroids from infected RMs compared to enteroids from uninfected (**Figure 3B**). Analysis of DEGs revealed downregulation of *ACE2* mRNA in the enteroids from infected RMs compared to enteroids from uninfected controls (Log_2_ fold-change= -1.91, *p* = 0.0000158, adjusted *p* = 0.00147), which was consistent with our qRT-PCR analysis. We also detected downregulation of genes such as *MME* (Membrane Metalloendopeptidase, Log_2_ fold-change= -1.69, *p* = 0.0000017, adjusted *p* = 0.00027), *ANPEP* (Alanine aminopeptidase, Log_2_ fold-change= -2.34*, p* = 0.0000081, adjusted *p* = 0.00091), and *ENPEP* (Glutamyl aminopeptidase, Log_2_ fold-change= -2.47, *p* = 0.0002332, adjusted *p* = 0.0002332), all of which are part of the RAS pathway (**Figure 3B**). The KEGG pathway analysis revealed a RAS pathway where all 4 genes (*ACE2*, *MME*, *ANPEP*, and *ENPEP*) were significantly enriched (*p* = 0.037).

*DPP4* (Dipeptidyl Peptidase 4), a receptor for Middle East respiratory syndrome-CoV, had also been suggested as an alternative receptor for SARS-CoV-2 (52). We observed a significant downregulation of *DPP4* expression in enteroids from infected RMs compared to uninfected controls (Log_2_ fold-change= -1.86, *p* = 0.0000009, adjusted *p* = 0.00018) using rlog transformed gene expression values (**Figure 3B**). As depicted in the heat map, three genes, namely *AGT*, *AGTR1* and *AGTR2* (*p* = 0.9779, *p* = 0.9292, *p* = 0.9552, respectively), demonstrated no significant changes in expression level between enteroids from infected RMs and uninfected controls (**Figure 3B**). Similarly, we were unable to detect any significant changes in *TMPRSS2* expression (Log_2_ fold-change= -0.040, *p* = 0.91599, adjusted *p* = 0.97548). Using raw gene counts, nonparametric Spearman correlation coefficient analysis showed a strong positive correlation between gene expression of *ACE2* and *DPP4* (*r*= 0.867, *p* = 0.0022, **Figure 3C**), *ACE2* and *ENPEP* (*r* = 0.806, *p* = 0.0072, **Figure 3D**) and *ACE2* and *ANPEP* (*r* = 0.867, *p* = 0.0022, **Figure 3E**) but there was no statistically significant correlation detected between *ACE2* and *MME* gene expression using raw read counts (*r* = 0.612, *p* = 0.0667, **Figure 3F**).

**Figure 4:**
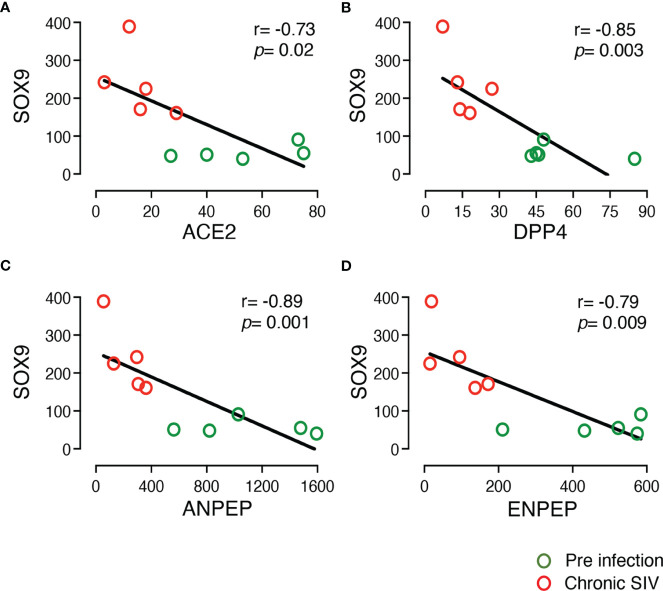
Negative correlation between the expression of *SOX9* and other important DEGs in the enteroid. The two-tailed Spearman’s correlation coefficient analyses between the expression of *SOX9* and *ACE2* **(A)**, *SOX9* and *DPP4* **(B)**, *SOX9* and *ANPEP* **(C)**, and *SOX9* and *ENPEP* **(D)** were performed using read counts obtained after transcriptomic analysis of the enteroids at pre (0 dpi) and chronic (180 dpi) infection for 5 subjects. *SOX*9 expression was upregulated in SIV infection compared to pre infection. The correlation and significant values are shown for each plot. Green and red open circles represent pre (0 dpi) and chronic (180 dpi) infection time points, respectively. *P value <*0.05 is considered statistically significant. Significant negative correlation was detected between *SOX9* and other important DEGs.

*SOX9* has been shown to inhibit ISC proliferation by regulating the WNT signaling pathway (53). We also observed a very significant upregulation of *SOX9* (Log_2_ fold-change = 1.88, *p* = 9.20E-10) in enteroids from chronically infected RMs compared to uninfected controls. Since *ACE2* seemed to act against *SOX9* during ISC proliferation, we examined the relationship between the expression of *ACE*2 and *SOX9*, which found a significant negative correlation (r= -0.73, *p* = 0.020; **Figure 4**). We have also detected negative correlation between gene expression of *SOX9* and *DPP4* and *ANPEP* and *ENPEP* (*p* < 0.05) (**Figure 4**).

Neuropilin-1 (NRP-1), a cell surface receptor for vascular endothelial growth factor (VEGF) and the class III Semaphorin family has been shown to facilitate SARS-CoV-2 cell entry and infectivity in different *in vitro* cell lines in the presence of ACE2 and TMPRSS2 receptor expression (54). NRP-1 is expressed by different immune cells including nonlymphoid cells like epithelial cells from the upper and lower intestines (55). Downregulation of *NRP-1* gene expression was detected following chronic SIV infection in jejunum enteroids compared to uninfected normal enteroids (Log_2_ fold-change = -1.83). However, the downregulated *NRP-1* expression in enteroids from chronically SIV infected RMs was not statistically significant (adjusted *p* = 0.139694) when compared to uninfected control enteroids.

### *HNF1A* Is a Key Inhibitor Regulator Based on Pathway and Upstream Regulator Analyses

**Figure 5:**
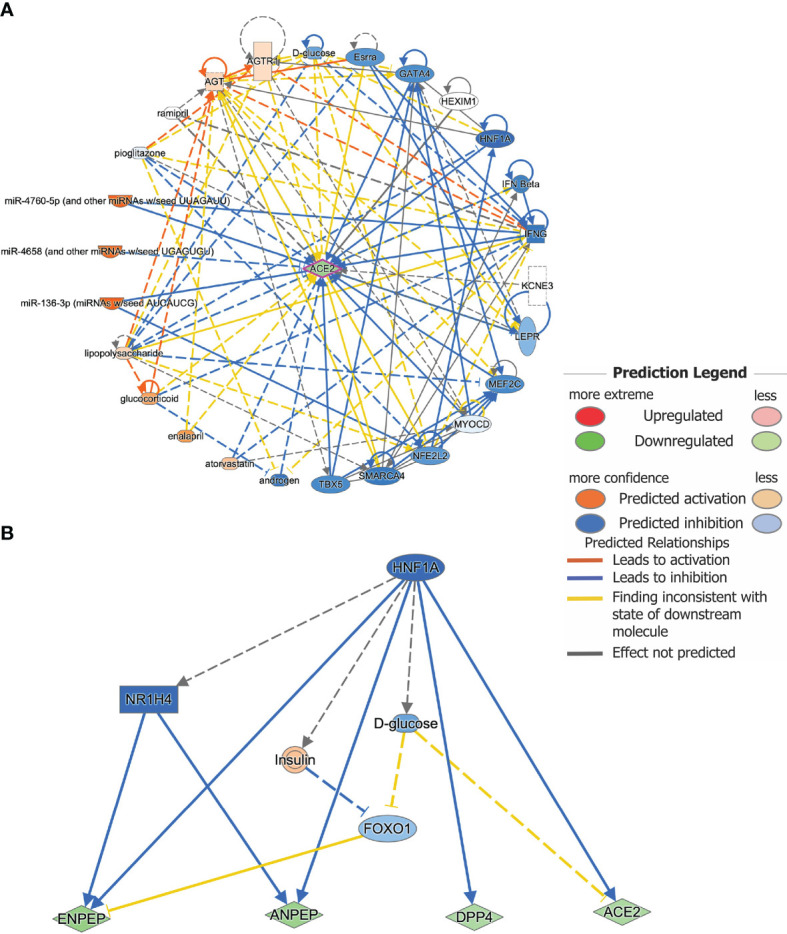
Upstream regulator analysis predicts key regulators for gene expression. **(A)** Upstream regulators for the gene *ACE2* as analyzed by IPA. A total of 26 predicted upstream regulators were identified as possible contributors to the change in ACE2 mRNA expression after infection. Known links between *ACE2* and the predicted upstream regulators are indicated. Different colors indicate the predicted relationships between the regulators and *ACE2* gene expression. **(B)** *HNF1A* was found to be a common upstream inhibitory regulator for *ACE2*, *DPP4*, *ENPEP*, and *ANPEP* gene expression.

IPA upstream regulator analysis identified 26 significant upstream regulators (*p* < 0.05) for *ACE2* based on all species (**Supplementary Table 3**). Among those upstream regulators, *HNF1A* was marked as a key inhibitory regulator (z-score = -4.271). Meanwhile, marked as activating regulators were miR-4658 (and other miRNAs w/seed UGAGUGU, z-scores = 2.111), *miR-4760-5p* (and other miRNAs w/seed UUAGAUU, z-scores = 2.887), and *miR-136-3p* (miRNAs w/seed AUCAUCG, z-scores = 2.887) (**Figure 5A**) (**Supplementary Table 3**). There were 12 significant upstream regulators in our data set, such as *HNF1A*, *AGT*, *SMARCA4*, etc. In particular, the significant upstream regulator *MEF2C* was also identified as a significantly downregulated DEG (log_2_ fold-change = -4.223, FDR = 0.0387) in RNA-seq data obtained from enteroids grown during pre and chronic infection (n=5). Upstream regulators *LEPR*, *MYOCD*, and *TBX5* were also downregulated (log_2_ fold-change = -3.152, -1.882, and -2.031, respectively). Meanwhile, upstream regulator *KCNE3* was upregulated in our data set. We also checked for any common upstream regulators and as expected, the transcription factor *HNF1A* was found to regulate transcription of all genes except *MME*, as analyzed in IPA (**Figure 5B**). This upstream regulation supported our correlation data, where all the DEGs except *MME* showed strong correlation with *ACE2* expression.

### No Significant Jejunum ACE2 Protein Expression Detected in SIV Infection

**Figure 6:**
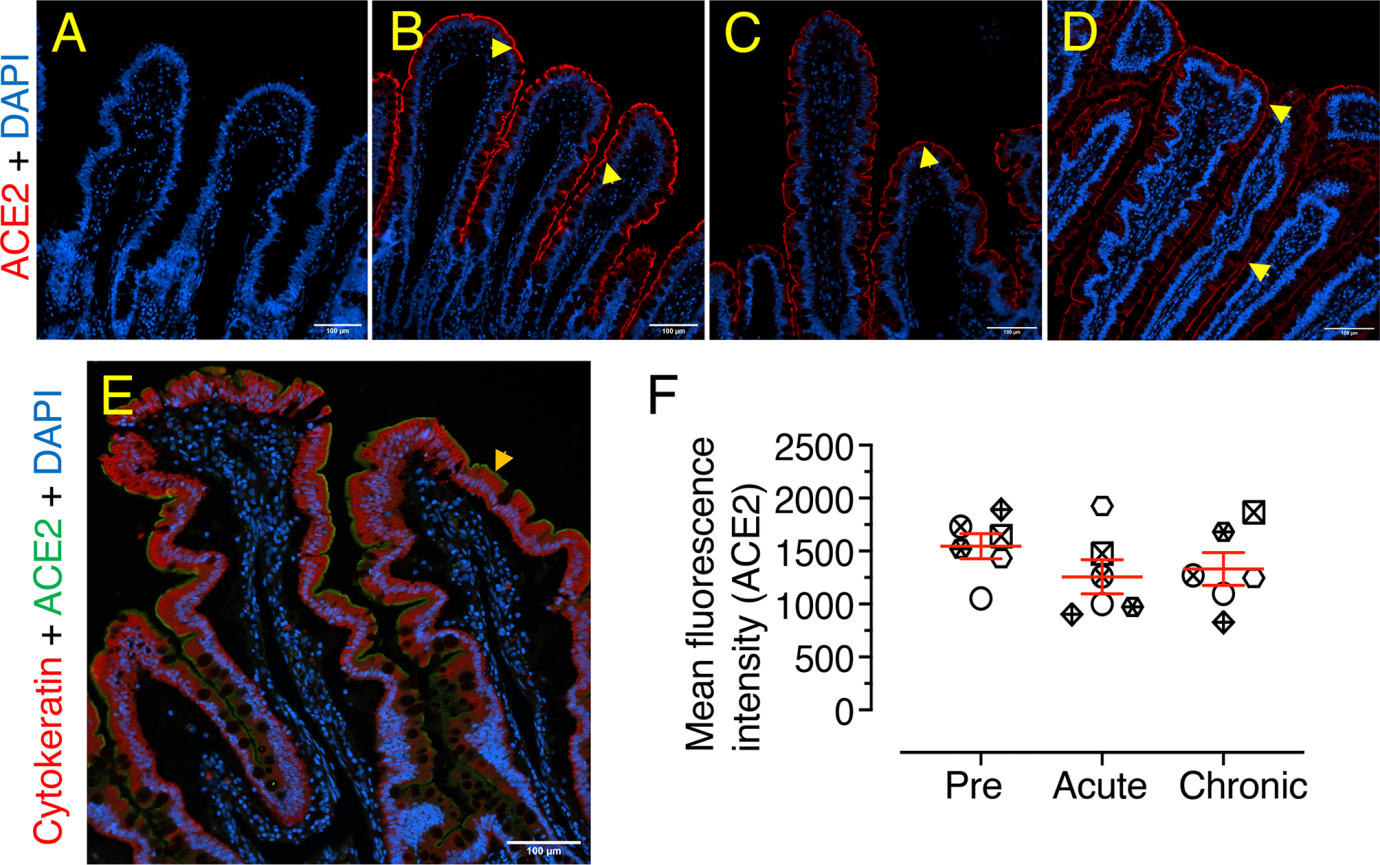
No significant changes in ACE2 protein expression detected in jejunum during SIV infection. Representative isotype control for ACE2 showing the absence of nonspecific background staining (**A**, KM05). Representative immunofluorescence images of ACE2 expression detected in RM KM05 during pre **(B)**, acute (21 dpi, **C**), and chronic (180 dpi, **D**) infection. Expressions of ACE2 proteins in the jejunum epithelium are shown by yellow arrows. **(E)** Co-localization of ACE2 (green) and cytokeratin (red), indicated by orange arrow at the epithelial barrier, showed ACE2 expression only at the intestinal brush border. DAPI stains the cell nucleus. **(F)** Scatter plots (indicating mean ± SE) of ACE2 immunofluorescence pixel values for pre, acute (21 dpi), and chronic (180 dpi) infection (n=6). An average of 20-23 regions of interest (20X objective) was randomly selected from villi from each animal to quantify ACE2 expression, and the mean intensity of these regions for each individual is represented by each point on the scatter plot. Each animal represents a different shape.

Significantly decreased ACE2 gene expression in infected RMs compared to uninfected controls underscores the importance of ACE2 protein expression in jejunum tissues during SIV infection. Jejunal tissue from 6 subjects (3 male and 3 female) were collected at three longitudinal data points (pre, acute, and chronic infection) (**Table 1**). ACE2 staining in the jejunum tissues revealed robust expression as well as localization at the brush border of the entire surface of each intestinal villus (**Figures 6A–D**). The tissue sections were also stained with both ACE2 and cytokeratin antibodies, which showed that the ACE2 expression was actually present in epithelium (**Figure 6E**). To determine the impact of infection on ACE2 expression, we analyzed the expression of ACE2 from each individual and calculated the mean fluorescence intensity. We noted reduced fluorescence intensity during acute (mean ± SE: 1257 ± 160.1) and chronic (1331 ± 155.8) compared to pre infection (1545 ± 118.3) (**Figure 6F**). However, these changes were statistically insignificant between any of the time points, as well as the comparison of ACE2 protein expression between male and female RMs was not statistically significant (**Supplementary Figure 5**).

### Significantly Increased AGTR2 Expression in Jejunum During Chronic Infection

**Figure 7:**
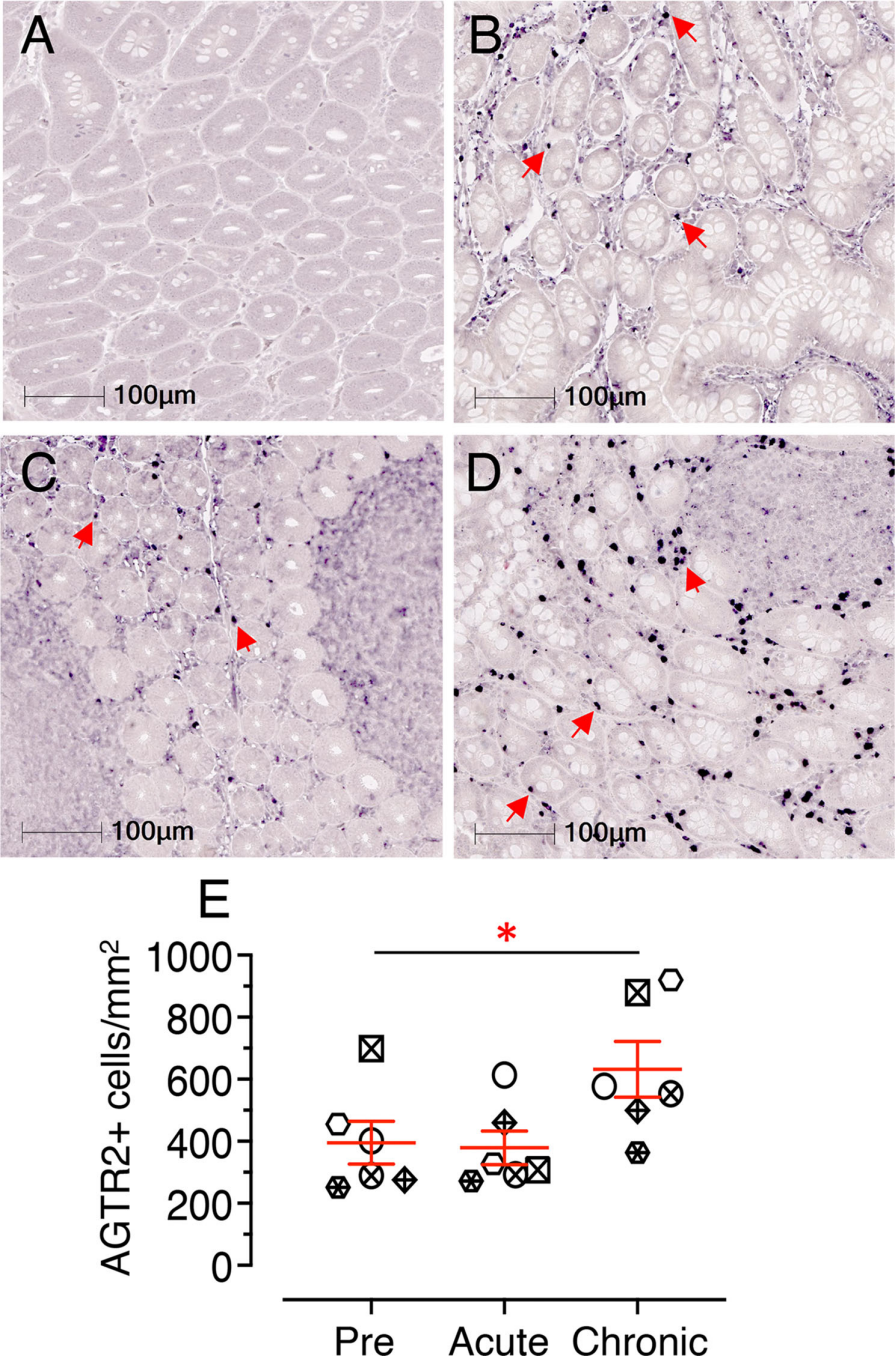
Chronic infection induces significant increase in jejunum AGTR2 expression. Representative Isotype control for AGTR2 showing the absence of nonspecific background staining (**A**, KP54). Representative immunofluorescence images of AGTR2 expression detected in a RM (KP54) pre (0 dpi, **B**), acute (21 dpi, **C**), and chronic (180 dpi, **D**) infection. Expressions of AGTR2 proteins in the lamina propria region of the jejunum are shown by red arrows. Note there was a significant number of AGTR2+ cells detected during chronic infection in this animal. **(E)** A scatter dot plot representing the mean frequency of AGTR2+ cells/mm^2^ of jejunum tissue in 6 individuals is shown at pre, acute, and chronic infection time points. The horizontal line denotes the mean frequencies (± SE) of each group. Twenty equal-size regions of interest were manually drawn in the lamina propria and analyzed with the multiplex IHC module of the Halo software. The mean cell counts of these regions for each individual is represented by each point on the scatter plot. Statistically significant differences between groups as calculated by Bonferroni analysis are indicated with asterisks (*p < 0.05). Each animal represents a different shape.

Jejunum tissue sections from 6 subjects (3 male and 3 female) were stained for IHC to detect AGTR2+ cells during pre, acute, and chronic SIV infection. The majority of AGTR2+ cells were localized in the lamina propria region (**Figures 7A–D**). A small number of AGTR2+ cells were also detected in epithelial cells **(** **Supplementary Figure 6A, B**). To determine the impact of SIV infection on AGTR2 expression, AGTR2+ cells were quantified in jejunum. A significant increase in AGTR2+ cells was detected during chronic (mean ± SE: 632 ± 90, *p* = 0.015) compared to pre infection (395 ± 69) (**Figure 7E**). There was no significant difference in the number of AGTR2+ cells between acute (379 ± 54) and pre infection time points nor between male and female RMs (**Supplementary Figure 6C** **)**.

### Decreased Jejunum Villi TMPRSS2 Protein Expression Detected During SIV Infection

**Figure 8:**
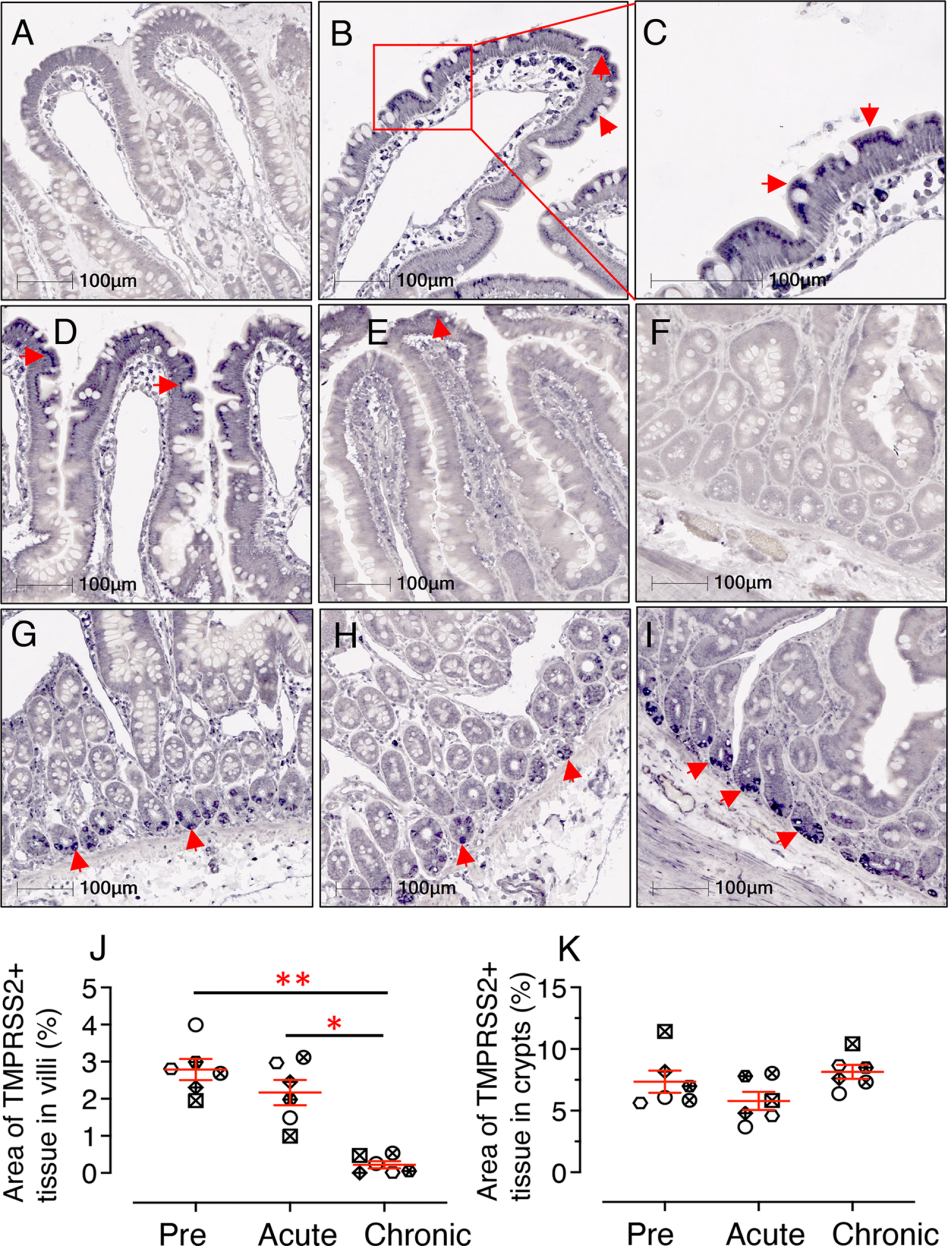
SIV infection significantly downregulates jejunum villi TMPRSS2 expression. Representative isotype control for TMPRSS2 showing the absence of nonspecific background staining in jejunum villi (**A**, RM JK56). Representative immunohistochemistry (IHC) images of TMPRSS2+ epithelium are shown for JK56 during pre infection (**B**, 10x objective; **C**, 20x objective), acute (21 dpi, and **D**), chronic (180 dpi, **E**). **(C)** Jejunum villi at 20x objective magnification shows the expression of TMPRSS2 as a transmembrane protein just beneath the epithelial brush border. **(F)** Representative isotype control for TMPRSS2 showing the absence of nonspecific background staining in jejunum crypts (KA76). Representative IHC images of TMPRSS2+ epithelium during pre **(G)**, acute **(H)** and chronic **(I)** infection are shown in jejunum crypts for RM KA76. Note, unlike in villi, TMPRSS2 is strongly expressed in the jejunum crypts during chronic infection **(E, I)**. The red arrows depict localization of TMPRSS2. Scatter plots showing percentage area of TMPRSS2+ epithelium in villi **(J)** and crypts **(K)** during pre, acute, and chronically infection. The larger horizontal line denotes mean frequencies (± SE) for each category (n=6). TMPRSS2 expression in the villi epithelium and crypts was quantified by gating ROI. An average of 20 and 30 fields (10x objective) for villi and crypts, respectively, was used to quantify TMPRSS2+ epithelium from each animal. Each point on the scattered plot represents mean TMPRSS2+ area for each individual. Each symbol represents individual macaque in each plot. Asterisks indicate statistical differences between stages of infection as calculated by Bonferroni analysis (*p < 0.05; **p < 0.01).

Similar to earlier analyses, 6 individuals were selected to quantify TMPRSS2 protein expression in jejunum tissues using IHC staining during pre, acute, and chronic infection. Expression of TMPRSS2 protein was more pronounced at the tip of the villi and gradually reduced toward the base (**Figures 8A–D**). Since TMPRSS2 is a transmembrane protein, it was localized just below the epithelial outer membrane (**Figure 8C**). Expression patterns during pre and post infection varied between villi (**Figures 8A–E**) and crypt regions (**Figures 8F–I**). Hence, TMPRSS2 expression was quantified separately for each region. In villi, a significant decrease in TMPRSS2 expression was observed during chronic infection (mean ± SE: 0.22 *±* 0.09%) compared to pre (2.79 *±* 0.28%, *p* = 0.001) and acute (2.17 *±* 0.34%, *p* = 0.010) (**Figure 8J**). In crypts, which are less exposed, no significant change in TMPRSS2 expression was observed during infection compared to pre infection time points (pre: 7.35 ± 0.90%; acute: 5.78 ± 0.73; and chronic: 8.14 ± 0.56) (**Figure 8K**). TMPRSS2 expression in jejunum villi epithelium between male and female RMs was not statistically significant either (**Supplementary Figure 7**).

### Loss of Jejunum CD4+ T-Cells Correlates With Increased AGTR2 and Decreased TMPRSS2 Expression During Infection

**Figure 9:**
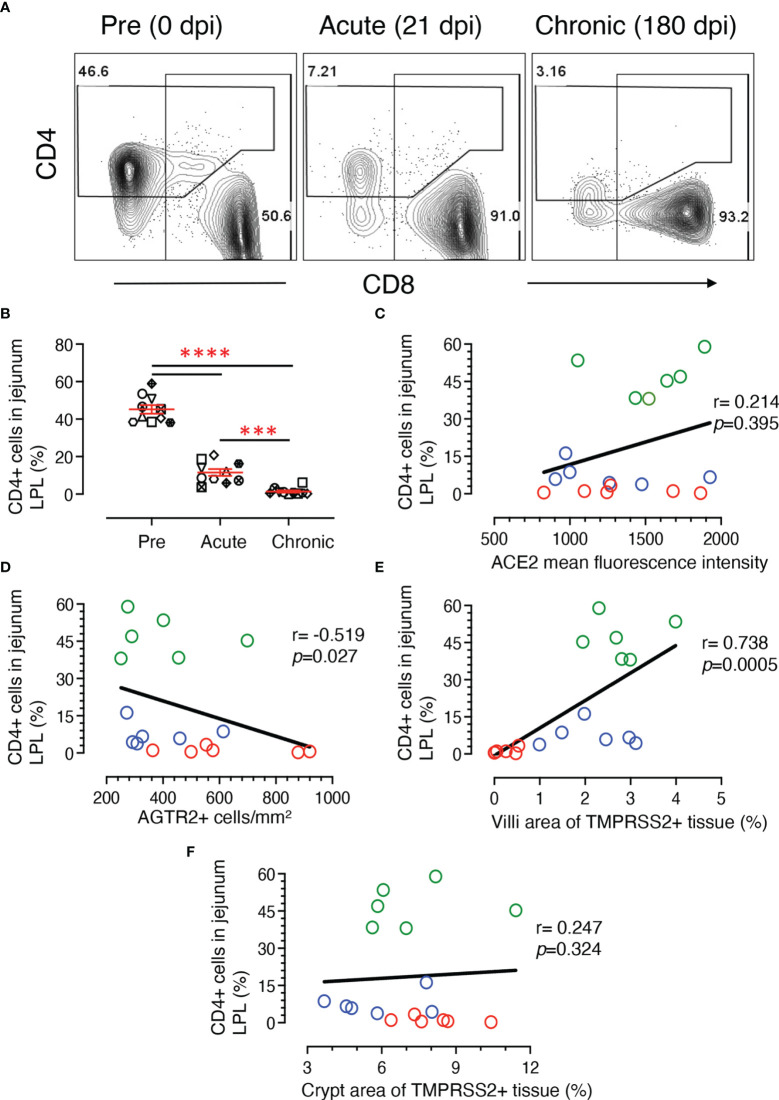
Loss of jejunum lamina propria CD4+ T-cells correlates with increased AGTR2 and decreased TMPRSS2 expression during infection. **(A)** Representative contour plots showing loss of jejunum lamina propria CD4+ T cells during acute (21 dpi) and chronic (180 dpi) compared to pre (0 dpi) infection time point. In each plot, the percentage of CD4 and CD8+ T cells are shown in the top left and lower right position, respectively. **(B)** Scatter plots showing percentages of CD4+ T cells (mean ± SE) from jejunum LPL with significant loss of CD4+ T cells during acute and chronic infection compared to pre time points (n=10). Each animal is symbolized by a different shape. Asterisks indicate statistical differences between stages of infection as calculated by Bonferroni analysis (***p < 0.001 and ****p < 0.0001). A two-tailed Spearman’s correlation coefficient analysis between percentages of CD4+ and ACE2 mean fluorescence intensity **(C)**, percentage of CD4+ and AGTR2+ cells **(D)**, CD4+ and TMPRSS2+ in villi **(E)**, and CD4+ and TMPRSS2+ in crypts **(F)** is shown for 6 individuals at pre, acute, and chronic infection. Green, blue, and red open circles denote pre, acute, and chronic infection time points, respectively. Significant negative and positive correlation was detected between percentages of CD4+ T cells and AGTR2+, and TMPRSS2+ in villi, respectively.

Depletion of early CD4+ T cells in SIV/HIV infection contributes to disease pathogenesis and accelerates mucosal inflammation and subsequent microbial translocation. A significant loss of jejunum CD4+ T cells was detected in infected RMs where CD4+ population decreased more than 6-fold during acute infection and remained low during chronic infection (**Table 1** and **Figure 9A** **).** In summary, there was a significant decrease in CD4+ T cell population during acute (mean ± SE: 11.5 ± 1.8%; *p* < 0.0001) and chronic (1.4 ± 0.6%; *p* < 0.0001) compared to pre infection (45.2 ± 2.3%) (**Figure 9B**). The reduction in jejunum CD4+ T cells from the acute to chronic stage was also statistically significant (*p* = 0.0007) (**Figure 9B**).

Using data from 6 subjects, two-tailed Spearman’s correlation coefficient analyses were performed at pre, acute, and chronic infection time points for a series of pairwise comparisons between jejunum CD4+ T cell percentages and jejunum ACE2 intensity, jejunum CD4+ frequency and area of TMPRSS2+ epithelium, and jejunum CD4+ frequency and AGTR2+ cells. Jejunum CD4+ T cell percentages showed no significant correlation with ACE2 expression (r = 0.214, *p* = 0.395) (**Figure 9C**). However, a significant negative correlation was shown between CD4 and AGTR2+ cells in jejunum (r = -0.519, *p* = 0.027) (**Figure 9D**). Jejunal CD4+ T cell percentages showed a significant positive correlation with TMPRSS2 expression in villi epithelium (r = 0.738, *p* = 0.0005) (**Figure 9E**). Unlike at the surface, TMPRSS2 expression in crypts had no significant correlation with that of jejunal CD4+ T cell percentages (r = 0.247, *p* = 0.324) (**Figure 9F**).

### Plasma MCP-1 Expression Is Negatively Correlated With Jejunum LPL CD4+ and Villi TMPRSS2 Expression

**Figure 10:**
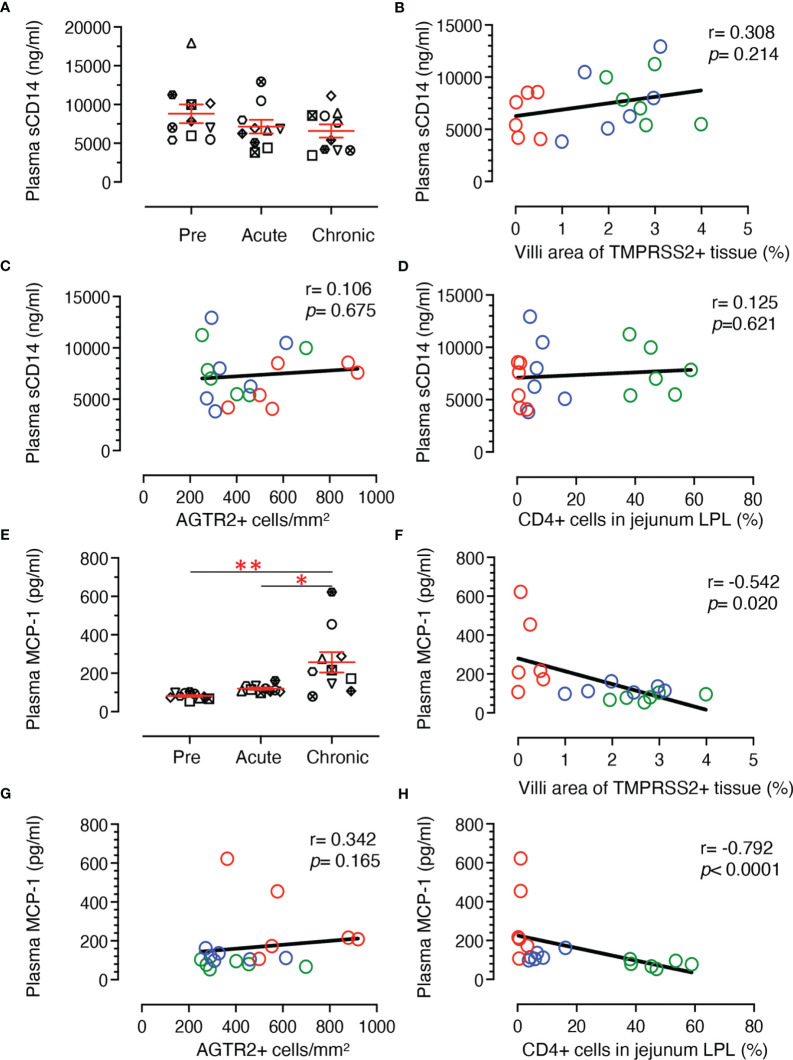
Plasma sCD14 and MCP-1 concentration and its correlation with jejunum CD4+, AGTR2+, and TMPRSS2+ expression in jejunum tissue. **(A)** Scatter plots showing plasma concentration of sCD14 (ng/mL) at pre (0 dpi), acute (21 dpi), and chronic (180 dpi) SIV infection. The error bars represent the mean ± SE for each time point (n=10). A two-tailed Spearman’s correlation coefficient analysis between plasma sCD14 concentration and TMPRSS2+ in villi **(B)**, plasma sCD14 level and AGTR2+ cells **(C)**, and plasma sCD14 level and percentages of CD4+ in jejunum LPL **(D)** is shown for 6 subjects at pre, acute, and chronic infection. **(E)** Scatter plots showing plasma concentration of MCP-1 (pg/mL) at pre, acute, and chronic SIV infection. The error bars represent the mean ± SE for each time point (n=10). Asterisks indicate statistical differences between stages of infection as calculated by Bonferroni analysis (*p < 0.05 and **p < 0.005). A two-tailed Spearman’s correlation coefficient analysis between plasma MCP-1 concentration and TMPRSS2+ in villi **(F)**, plasma MCP-1 level and AGTR2+ cells **(G)**, and plasma MCP-1 level and percentages of CD4+ in jejunum LPL **(H)** is shown for 6 subjects at pre, acute, and chronic infection. Significant negative correlations were detected between MCP-1 levels and TMPRSS2+ in villi and percentages of CD4+ T cells. Each symbol represents individual macaque. Green, blue, and red open circles denote pre, acute, and chronic infection time points, respectively.

Soluble CD14 (sCD14) is an indirect measurement of monocyte activation and microbial translocation as has been demonstrated in earlier HIV studies (56, 57). Plasma sCD14 levels did not change significantly during SIV infection in our longitudinal study with 10 RMs (**Figure 10A**, mean ± SE ranged from 8802 ± 1206, 7142 ± 877, and 6589 ± 845 ng/ml for pre, acute and chronic time points, respectively). Two-tailed Spearman’s correlation coefficient analyses between sCD14 plasma levels and % of TMPRSS2+ tissue in villi (**Figure 10B**), sCD14 concentration and AGTR2+ cells (**Figure 10C**), and plasma sCD14 level and CD4+ T cells (**Figure 10D**) at pre, acute, and chronic infection time points indicated no significant correlation between sCD14 and AGTR2+/TMPRSS2+/CD4+ T cells. Monocyte chemoattractant protein-1 (MCP-1) is one of the key chemokines that plays an important role in regulating migration and infiltration of monocytes/macrophages (58) and was quantified in the longitudinal study using 10 subjects infected with SIV. A significant upregulation of plasma MCP-1 concentration was detected during chronic stage (mean ± SE, 257 ± 53 pg/mL) compared to pre (mean ± SE, 81 ± 5 pg/mL; p < 0.001) and acute infection (mean ± SE, 119 ± 6 pg/mL; p < 0.01) time points (**Figure 10E**). Two-tailed Spearman’s correlation analyses demonstrated a significant negative correlation between plasma MCP-1 levels and TMPRSS2 expression in villi epithelium (r = 0.542, *p* = 0.020, **Figure 10F**) and between plasma MCP-1 levels and the CD4+ T cell percentages from jejunum LPL (r = 0.792, *p* < 0.001, **Figure 10H**). No statistically significant correlation was detected between plasma MCP-1 level and the frequency of AGTR2+ cells in jejunum (r = 0.342, *p* = 0.165) (**Figure 10G**)

### Dynamics of Plasma ACE2 Level

**Figure 11:**
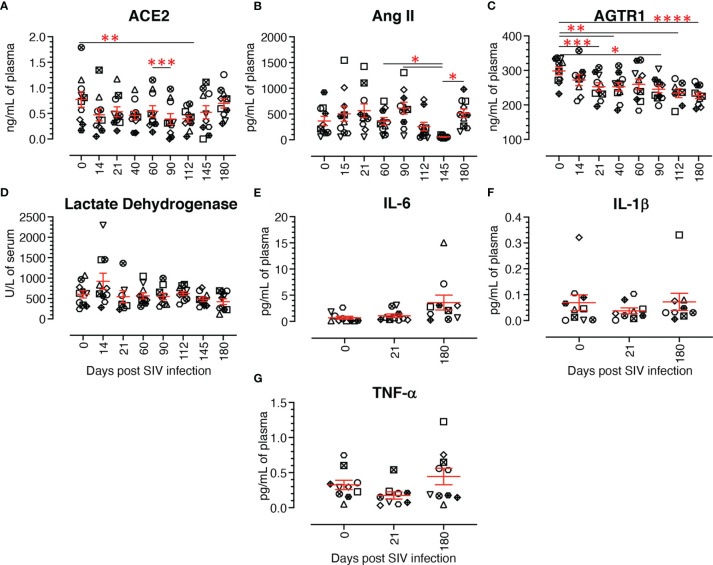
Dynamics of different renin angiotensin system proteins, lactate dehydrogenase, and cytokine production in plasma/serum. **(A)** Plasma concentration of ACE2 (ng/mL) at different time points of infection. The plasma ACE2 gradually decreased from acute to chronic infection with the lowest concentration at 90 dpi, then recovered during the late chronic stage of infection. **(B)** Plasma concentration of Angiotensin II (Ang II, pg/mL) at different time points. Though not significant, post infection showed a slight increase of Ang II compared to the pre infection time point, except at 112 and 145 dpi. **(C)** Plasma concentration of angiotensin II receptor 1 (AGTR1) (ng/mL) during infection. A gradual decrease of plasma AGTR1 was detected from 14 dpi onward. **(D)** Serum LDH activity (U/L) in infected RMs and uninfected controls were evaluated using a Beckman Coulter AU 480 analyzer. No significant changes in serum LDH activity were detected across the different time points. Plasma IL-6 **(E)**, IL-1β **(F)**, and TNF-α **(G)** concentrations (pg/mL) at pre and post infection time points were evaluated by U-plex biomarker NHP multiplex assay. No significant changes were detected at any time points for any of the proinflammatory cytokines tested. The error bars represent the mean ± SE for each time point (n=10). Each symbol represents individual macaque in each plot. Asterisks indicate statistical differences between time points, as calculated by Bonferroni for ACE2 and Ang II and Tukey-Kramer for AGTR1 (*p < 0.05; **p < 0.01; ***p < 0.001 and ****p < 0.0001).

Circulatory plasma ACE2 protein levels from 10 subjects at pre and post infection time points were quantified by ELISA to determine whether changes in ACE2 expression were tissue specific. The ACE2 concentration during pre infection ranged from 0.17 ng/mL to the highest 1.79 ng/mL (mean ± SE: 0.77 ± 0.15 ng/mL). After infection, the ACE2 level went down as low as 0.39 ± 0.11 ng/mL at 90 dpi. ACE2 level reverted after 90 dpi, reaching an almost normal concentration of 0.70 ± 0.09 ng/mL at 180 dpi. Plasma ACE2 concentrations remained low during most of the post infection time points compared to pre infection (**Figure 11A**), but ACE2 levels at post infection time points were not statistically significant except at 112 dpi (*p* = 0.003).

### Dynamics of Plasma Ang II Quantification

Circulatory plasma Ang II levels in 10 subjects during pre and post infection were measured by ELISA. Ang II plasma concentration during pre infection ranged from 59.7 to 921.3 pg/mL (mean ± SE: 365.3 ± 89.6 pg/mL). After infection, Ang II concentration varied widely at 14 dpi (ranging from 33.5 to 1543.7 pg/mL) (**Figure 11B**). No significant changes in Ang II concentration were detected between pre and any of the post infection time points. Ang II levels remained higher at most of the post infection time points, except at 112 and 145 dpi, compared to pre infection (**Figure 11B**). The highest and lowest plasma Ang II levels were detected at 90 dpi (mean ± SE: 614.9 ± 114.3 pg/mL) and 145 dpi (mean ± SE: 54.9 ± 5.9 pg/mL), respectively (**Figure 11B**). Though we detected a negative correlation between ACE2 and Ang II plasma concentration in infected RMs, it was not statistically significant.

### Significant Changes in Plasma AGTR1 Concentrations During SIV Infection

Plasma AGTR1 concentrations were measured in 10 subjects at pre and post infection time points. Before infection, AGTR1 levels ranged from 232.1 to 336.2 ng/mL of plasma (mean ± SE: 298.6 ± 11.5 ng/mL). AGTR1 level reduced during infection with the lowest at 180 dpi (mean ± SE: 227.3 ± 8.8 ng/mL) (**Figure 11C**). We also observed a significantly reduced AGTR1 plasma level compared with pre infection at 21 dpi (*p* = 0.0006), 40 dpi (*p* = 0.0086), 90 dpi (*p* = 0.0220), 112 dpi (*p* = 0.0021), and 180 dpi (*p* < 0.0001).

### No Significant Changes in Serum LDH Activity During SIV Infection

A significantly higher level of serum LDH has been found to be correlated with HIV progression and the prevalence of opportunistic infections in HIV infected patients (59, 60). We therefore wanted to study the dynamics of LDH level in infected RMs, as well as determine its association with ACE2 level. LDH concentration was measured in serum during SIV infection in our longitudinal study with 10 RMs. Serum LDH concentration from uninfected RMs was 602.9 ± 89.2 U/L (mean ± SE) and it remained stable throughout the period of the study with highest concentration at 14 dpi (mean ± SE: 923.5 ± 197.0 U/L) and lowest at 180 dpi (mean ± SE: 423.0 ± 70.3 U/L) (**Figure 11D**). No significant difference in LDH level was observed between any of the time points in the study. A correlation analysis between serum ACE2 and LDH levels detected a negative correlation, however it was not statistically significant.

### No Significant Changes in Plasma Inflammatory Cytokines Level

Plasma IL-6, IL-1β, and TNF-α concentration was measured during pre, acute (21 dpi), and chronic infection (180 dpi). Mean concentrations of IL-6, IL-1β, and TNF-α pre infection were 0.69 ± 0.27 pg/mL, 0.07 ± 0.03 pg/mL, and 0.32 ± 0.06 pg/mL (mean ± SE), respectively (**Figures 11E–G**). IL-1β concentration reduced slightly during acute (0.04 ± 0.01 pg/mL) and reverted back to normal during chronic infection (0.07 ± 0.03 pg/mL) (**Figure 11F**). Similar to IL-1β, TNF-α level was reduced during acute (0.17 ± 0.05 pg/mL) and reverted back to normal during chronic infection (0.44 ± 0.12 pg/mL) (**Figure 11G**). In contrast, compared to pre infection, IL-6 concentration gradually increased during acute (1.08 ± 0.34 pg/mL) and chronic infection (3.59 ± 1.42 pg/mL) (**Figure 11E**). However, no significant differences in cytokine levels were detected among different time points. The correlation analysis between inflammatory cytokines and ACE2, TMPRSS2 and AGTR2 revealed no significant correlation.

### No Significant Changes in Lung ACE2 Expression After Infection

**Figure 12:**
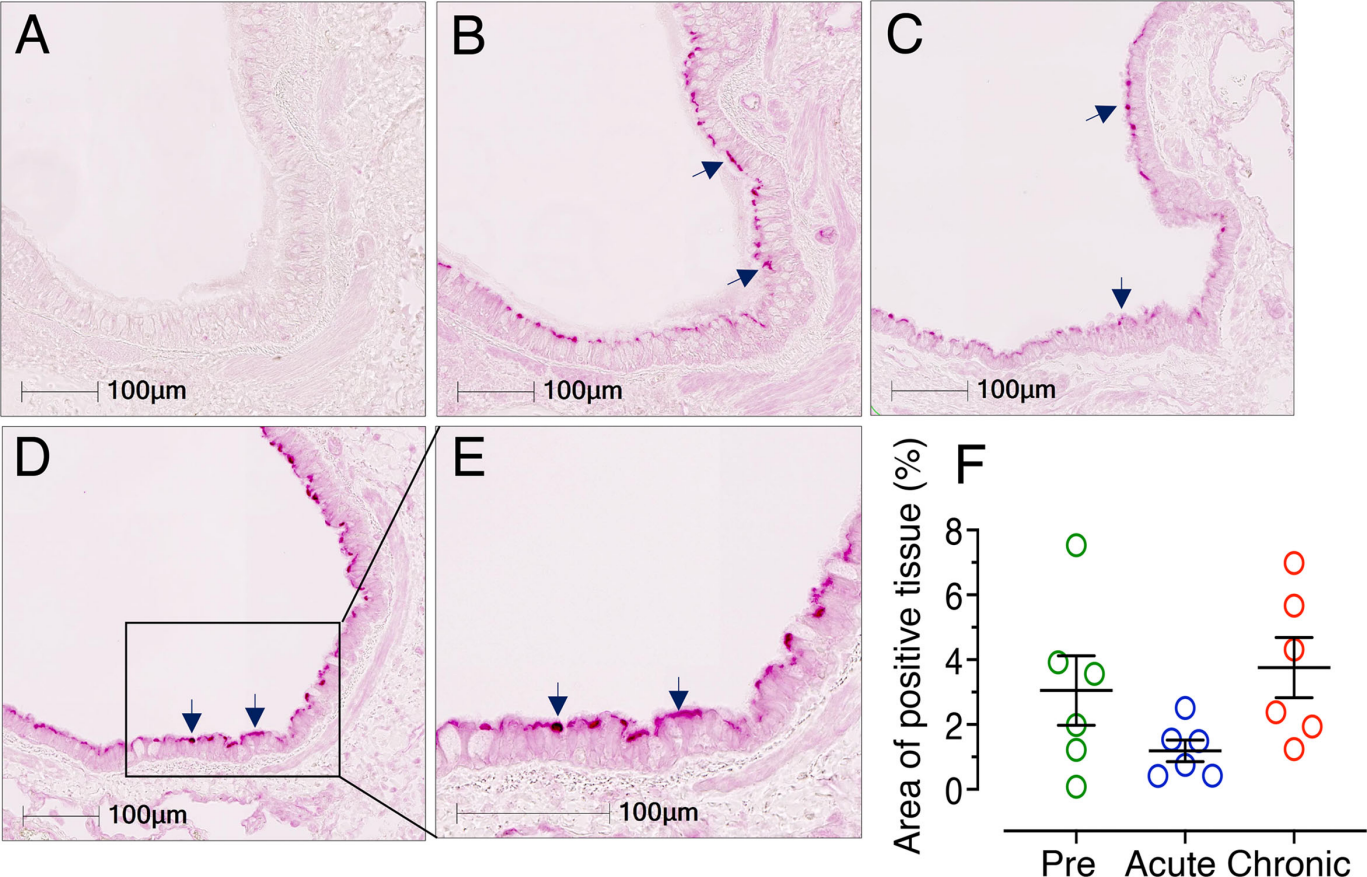
Reduced ACE2 expression detected in lung during acute infection. Representative isotype control for ACE2 showing the absence of nonspecific background staining in lung (**A**, RM FF25, 10x objective). Representative immunofluorescence images of ACE2 expression detected during pre (**B,** RM FF25), acute (21 dpi in EM64, **C**), chronic (**D**, 180 dpi in KP54, 10x objective), and chronic (**E**, 180 dpi in KP54, 20x objective) SIV infection. Expressions of ACE2 proteins in the bronchiole epithelium are shown by black arrows. **(F)** Scatter plots (with means ± SE) showing percentages of ACE2 positive tissue per total ROI in the bronchiole epithelium. Each point represents the average percentage of ACE2 positive tissue area from different bronchioles of each individual. ACE2 expression was reduced during acute infection, but the values were not statistically significant. ACE2 expression reverted to normal levels in chronic infection and remained higher compared to acute infection (n=6).

Considering the importance of the COVID-19 pandemic, we extended our study to examine levels of ACE2 protein expression in lung tissue to understand the degree of risk to HIV patients in acquiring SARS-CoV-2 infection. ACE2 expression was determined by IHC in cohorts of uninfected, acutely infected (21 dpi), and chronically infected subjects (180 dpi) (n=6). A clear and distinct ACE2 distribution in the bronchiole epithelium was observed (**Figure 12**). Therefore, the bronchiole epithelium was designated as our ROI for determining ACE2 expression in every bronchiole in each tissue section. ACE2 expression in acutely (mean ± SE, 1.19 ± 0.33%, *p* = 0.415) and chronically infected RMs (mean ± SE: 3.75 ± 0.93%, *p* = 0.142) was not statistically significant compared to pre infection (mean ± SE: 3.05 ± 1.07%) (**Figure 12F**). Overall, ACE2 expression at 21 dpi decreased in both lung and jejunal tissues (**Figures 6**, **12**) and reverted back to normal during chronic infection in lung tissues.

## Discussion

The role of different RAS components is well-established in many physiological disease conditions such as hypertension, congestive heart failure, obesity, hepatic complications, kidney disease, diabetes, ocular diseases, and neurological disorders including Parkinson’s disease, Alzheimer’s disease, and multiple sclerosis (1). Emerging evidence from the COVID-19 pandemic suggests that ACE2 and TMPRSS2, key players in RAS, are major receptors for SARS-CoV-2 spike proteins (61, 62). ACE2 is highly expressed in the intestinal epithelium where SARS-CoV-2 infection induces increased production of intestinal proinflammatory cytokines, intestinal infection, and the release of viral RNA through feces (61). HIV associated gastroenteropathy is initiated by early loss of CD4 T cells, loss of mucosal barrier, lack of anti-inflammatory response, increased microbial translocation, and chronic immune activation. Recent studies demonstrated a 1.5 to 3-fold increase in mortality in HIV patients with COVID-19 coinfection compared to HIV infection alone (63–65). Out of 38 million HIV infected living people, 73% of them have access to antiretroviral therapy (ART). The majority of those HIV patients not receiving ART reside in the Sub-Saharan Africa (66). Recent studies suggest that SARS-CoV-2 infection in HIV infected patients, those are not treated with ART or has low CD4 count, have increased severity of COVID-19 compared to those who are negative for HIV (67–69). Moreover, COVID-19 outbreak has also disrupted the ongoing HIV treatment and prevention program worldwide, which may eventually increase the percentage of HIV infected people without ART. Currently several antiretroviral drugs including tenofovir, darunavir, and maraviroc are being investigated for their potential usage for the prevention of SARS-COV-2 replication (70). Therefore, it’s important to understand the effect of HIV infection on ACE2 receptors expression as well as the expression of different RAS proteins and genes in jejunum tissue and peripheral circulation, which might have a major impact on COVID-19 transmission and pathogenesis. In this study we have used RM-SIV model to address some of these questions which is practically impossible to answer in HIV infected subjects without ART during acute and chronic infection. We studied the expression pattern of ACE2, TMPRSS2, and other RAS related proteins and genes in blood, lung, and jejunum tissues to understand their role in HIV/SIV pathogenesis and possible impact on SARS-CoV-2 coinfection. This study was subject to some limitations. The animal sample size was modest; some of the data variation could have been avoided by increasing it. However, this study has longitudinal strength, using jejunum and plasma samples obtained throughout the course of SIV infection.

Under homeostatic conditions, we observed a very strong expression of ACE2 in the jejunum epithelium, as reported elsewhere (71). As in intestinal inflammatory diseases, gut inflammation is one of the most important clinical manifestations of SIV/HIV infection. Consistent with various gut inflammatory conditions (32, 33, 35), *ACE2* mRNA expression was significantly downregulated during SIV infection. The negative correlation detected between *ACE2* and *IL-6* gene expression in the jejunum suggested a negative impact of inflammation on *ACE2* expression, or vice-versa. This may induce gut inflammation directly or indirectly, as reported for other intestinal inflammatory diseases (33, 51, 72, 73). Earlier studies using *ACE2* knockout mice have addressed the supplementary role of ACE2 in regulating gut microbiome ecology, intestinal inflammation, innate immunity, and amino acid homeostasis (16, 74). *ACE2* downregulation due to SARS-CoV-2 infection enhances systemic inflammation in COVID-19 patients (15). Decreased *ACE2* has been shown to impede metabolism of Ang II into the beneficial peptide Ang 1-7, resulting in luminal AGTR1 activation and enhanced permeability, and in turn led to leaky gut syndrome (17). The mechanisms behind the loss of ACE2 in SIV infection could be different from those of SARS-CoV-2 infection. However, downregulation of ACE2 during SIV infection could also account for similar downstream clinical sequelae such as leaky gut, dysbiosis, and loss of epithelial homeostasis.

Reduced *ACE2* expression in enteroids grown from chronically infected RMs also suggests that SIV infection negatively impacts *ACE*2 gene expression in newly generated epithelial cells. *ACE*2 associated genes like *ANPE*P and *DPP4*, which were reported as markers of differentiated intestinal epithelial cells (75, 76), were significantly downregulated in enteroids from infected RMs compared to uninfected controls. This suggests that SIV infection contributes to the reduction of *ACE2*, which in turn reduces the expression of genes responsible for epithelial differentiation. A recent finding using *ACE* knock out (*ACE2* ^-/-^) mice also suggests that *ACE2* ^-/-^ enteroids have fewer LGR5+ cells and markedly increased permeability compared to ACE2^+/+^ enteroids (18). Our observation also supports a recent finding in which *DPP4*, *ANPEP*, and *ENPEP* were reported to be the top three genes correlated with *ACE2* expression *via* single cell RNA-seq analysis (77). *SOX9* is a transcription factor that suppresses and accelerates ISC proliferation and differentiation, respectively (53). Negative correlation between *ANPEP* and *SOX9* as well as *DPP4* and *SOX9* suggests that impairment of ISC proliferation and differentiation was also regulated by *SOX9* expression. *HNF1A*, a transcriptional regulator, regulates the expression of various important genes including those associated with intestinal cell differentiation and cadherin expression (78, 79). Our IPA analysis revealed various upstream regulators, among which *HNF1A* was found to be the most likely upstream negative regulator of *ACE2* and associated proteins’ mRNA transcription. Therefore, *HNF1A* may be a major regulator in intestinal *ACE2*, *ANPEP*, and *DPP4* expression, with a significant role in regulating intestinal homeostasis in SIV infection. *HNF4A*, a family member of *HNF1A*, was also identified as an upstream regulator of *ACE2* and *DPP4* gene expression in patients with inflammatory bowel disease (33). Our data from enteroids also suggest that SIV infection has minimal role in regulating *NRP-1* gene expression in intestinal epithelial cells. Further studies are needed to determine the protective role of *ACE2* and its associated genes/proteins in regulating intestinal homeostasis during SIV infection and pathogenesis.

*ACE2*, *ANPEP*, *DPP4*, and *ENPEP* have been well documented as potential receptors for human coronaviruses (77, 80). Multiple clinical and experimental studies have concluded that a deficiency of ACE2, induced by inhibition or deletion, may cause hypertension and even heart failure (6, 81, 82). Therefore, it is tempting to contemplate that SARS-CoV-2 mediated inflammation and pathogenesis may be exacerbated by the significant reduction in ACE2 expression in jejunum in the presence of intestinal inflammation (25, 83) and lack of intestinal homeostasis (39) during SIV/HIV infection. The concept of reduced ACE2 receptor expression on the cell surface leading to less SARS-CoV-2 infection does not seem to be supported, however, as no direct correlation was detected between ACE2 expression and the susceptibility and severity of SARS-CoV-2 infection.

Since earlier studies have presented mostly transcriptomic data, we expanded our study to include protein expression level in order to have a better understanding of the role of ACE2. Though the expression of ACE2 protein was shown to be in the same direction as mRNA expression in jejunum tissue and enteroids, the changes in ACE2 protein expression were statistically insignificant. This variation in the significance profile between mRNA and protein expression could be explained by various possible molecular and cellular events (84). It was reported that the protein levels were more conserved than the mRNA levels. Hence, differentially expressed mRNA had more fold-change than that of its protein counterpart, though both the expression profiles were in the same direction (85). Increased cellular proliferation and stress responses detected in epithelial cells during SIV infection (26) may also lead to the stronger differences from an ideal correlation between mRNA and protein expression (84). This variation could be avoided to some extent by increasing the sample size and performing single cell RNA-seq to precisely analyze ACE2 expression in the epithelial cells; further study may confirm ACE2 expression levels in the gut of SIV/HIV infection and intestinal inflammatory diseases.

TMPRSS2 cleaves the S protein and promotes SARS-CoV-2 entry in gut epithelial cells (86). TMPRSS2 was highly expressed in both villi and crypt epithelium in jejunum tissue. During chronic infection, TMPRSS2 expression was highly affected at the villi surface epithelium, but not in the crypt epithelium. Unlike protein expression, mRNA expression analysis from both enteroids and whole jejunal tissue has shown no difference in TMPRSS2 gene expression during infection. TMPRSS2 regulation and its biological function in jejunum tissues is not well defined. The presence of a significant, positive correlation between the frequency of jejunal CD4+ T cells and the expression of villi surface epithelium TMPRSS2 possibly indicates that during infection, loss of jejunal CD4 T cells and increased proinflammatory IL-6 cytokines have a major impact on the loss of surface epithelial TMPRSS2 protein compared to TMPRSS2 expressed in crypts. The impact on TMPRSS2 expression of the pathological tissue environment, but not the molecular event, is well supported by our transcriptomic analysis, where no changes in TMPRSS2 mRNA expression were noted in either jejunum tissue or enteroids. We were unable to detect any upregulation of plasma sCD14 during the course of chronic SIV infection nor any correlation between sCD14 plasma level and CD4 depletion, TMPRSS2 villi expression, or AGTR2+ cell frequency in jejunum tissue. However, a significant increase in plasma MCP-1 level during chronic infection might promote chronic proinflammatory responses and the accumulation and activation of monocytes/macrophages in the inflamed gastrointestinal tissue (87–91). The negative correlation of plasma MCP-1 concentration with TMPRSS2+ expression in jejunum villi as well as the loss of jejunum CD4+ lamina propria cells suggests a possible role of MCP-1 as a biomarker for the loss of jejunum CD4+ and TMPRSS2+ cells in villi epithelium as well as a marker of disease progression.

Ang II induces apoptosis through either AGTR1 or AGTR2 in a cell-type dependent signaling pathway. AGTR1 mediated apoptosis has been reported in coronary artery endothelial cells and transformed epithelial cells (92–94). AGTR2 activation induced apoptosis in some cells or cell lines, such as neurons, bladder cancer cells, and PC12W cells (95–97). Unlike other cells, AGTR2+ cells were also expressed in the majority of jejunum lamina propria cells. The strong and significant increase in AGTR2+ cells during chronic infection may contribute to disease progression by inducing intestinal epithelial cell apoptosis. Our assumption of apoptosis due to increased percentage of AGTR2+ cells is supported by an earlier study on an intestinal epithelial cell line (Caco-2) where the induction of epithelial cell apoptosis could be triggered by the activation of AGTR2 through GATA-6 and the Bax signaling pathway (27). AGTR2 has also recently been shown to be a potential coreceptor for SARS-CoV-2 entry in various human cells, including those of the central nervous system (98, 99). Therefore, an increased expression of AGTR2+ cells in gut tissues during infection may promote increased susceptibility to SARS-CoV-2 infection.

## Conclusion

**Figure 13:**
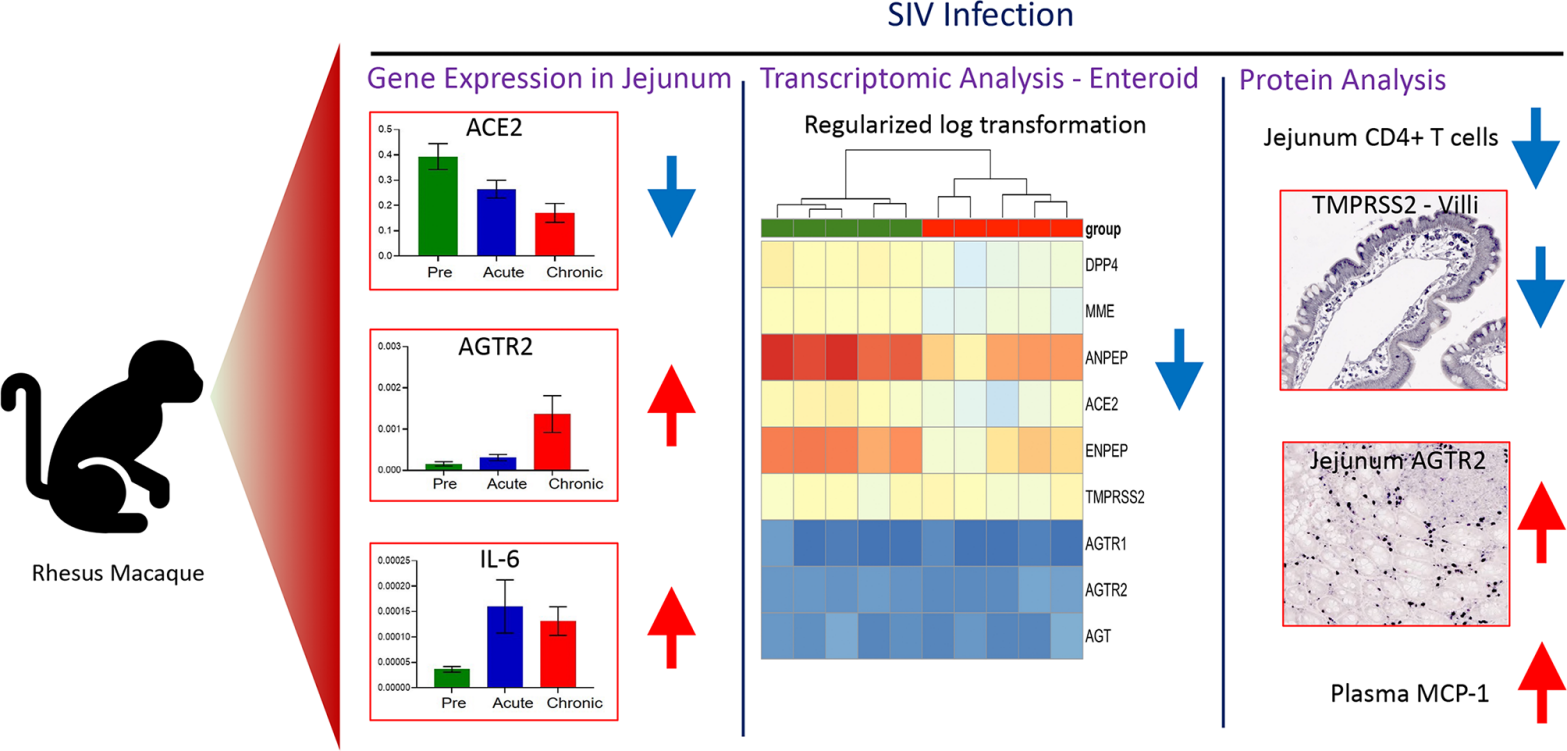
Schematic representation of the major findings. The study is stratified into three different groups: gene expression profiling of important RAS-associated proteins in Jejunum tissue during SIV infection in RM using real-time PCR; transcriptomic study of enteroids from pre and chronic SIV infected RM to identify differentially expressed gene involved in RAS; detection of RAS-associated protein expression in jejunum tissue. Gene expression analysis of jejunum tissue revealed significant differential expression of *ACE2*, *AGTR2*, and *IL-6* gene after SIV infection. RAS-associated DEGs identified in our global transcriptomic analysis were significantly downregulated in enteroids from crypts of SIV infected RMs. In addition to the loss of CD4+ LPL, the expression of TMPRSS2 protein in jejunum villi was also downregulated. Jejunum villi AGTR2 and plasma MCP-1 protein were upregulated in SIV infection. Blue and red arrows suggest a significant downregulated and upregulated genes or proteins, respectively.

Increased COVID-19 mediated mortality has been documented in immunosuppressed patients, yet HIV infection has yet to be identified as a potential comorbid condition in studies of hospitalized patients. There are conflicting reports on the relationship between HIV and SARS-CoV-2 infections with respect to mortality. We observed a significant downregulation of *ACE2*, *ANPEP*, *DPP*4, and *ENPE*P gene expression following SIV infection, though confirmation is required for ACE2 expression in protein level, suggesting that ACE2 mediated pathological changes may interfere with gut homeostasis along with loss of mucosal CD4+ T cells. A significantly decreased jejunal villi surface TMPRSS2 expression was also observed during SIV infection. In addition to the CD4+ T cell depletion, increased *IL-6* mRNA, MCP-1 and AGTR2 expression may signal inflammation, monocyte/macrophage accumulation and epithelial apoptosis in accelerating SIV pathogenesis. *HNF1A* transcription factor was predicted to be a key upstream negative regulator of *ACE2* and other gene expressions. Increased expression of AGTR2+ cells in jejunal tissue may act as a coreceptor in accelerating SARS-CoV-2 coinfection. A schematic diagram presenting the overall expression of major RAS associated proteins/genes during SIV infection is shown in **Figure 13**. Further studies are needed to understand the role of these RAS proteins in regulating different viral pathogenesis in the HIV and SARS-CoV-2 coinfection model.

## Data Availability Statement

The datasets presented in this study can be found in online repositories. The names of the repository/repositories and accession number(s) can be found below: SRA repository, accession number PRJNA799682 (https://www.ncbi.nlm.nih.gov/sra/PRJNA799682).

## Ethics Statement

The animal study was reviewed and approved by Tulane University IACUC.

## Author Contributions

The overall planning, direction and design of the experiment were carried out by BP. NB, KW, MS, and BP carried out animal scheduling, sample collection, sample processing and all experiments. BP, NB, and AD analyzed the flow data. NB and SG performed image acquisition and analysis. XC, QS, and BP performed RNA-seq data analysis and interpretation. PD and BP performed IHC and IF data interpretation. SS performed statistical analyses. KB provided scientific advice and performed manuscript review. NB and BP wrote the manuscript with inputs from all authors. All authors have reviewed and given approval to the final version of the manuscript.

## Funding

The study was supported by National Institutes of Health grant R01DK109883 (BP) and TNPRC base grant P51OD011104.

## Conflict of Interest

The authors declare that the research was conducted in the absence of any commercial or financial relationships that could be construed as a potential conflict of interest.

## Publisher’s Note

All claims expressed in this article are solely those of the authors and do not necessarily represent those of their affiliated organizations, or those of the publisher, the editors and the reviewers. Any product that may be evaluated in this article, or claim that may be made by its manufacturer, is not guaranteed or endorsed by the publisher.

## Supplementary Material

Supplementary Figure 1 The representative figures depict the gating strategy for region of interest (ROI) for ACE2 **(A)**, TMPRSS2 staining in villi **(B)** and crypts **(C)**. ROIs were manually drawn (in yellow or red lines) in the epithelium of randomly selected villi/crypts for the quantification of ACE2/TMPRSS2 intensity in epithelial cells.

Supplementary Figure 2 Bar plots of *ACE2* and associated genes expression in the jejunum tissue of uninfected RM as determined by qRT-PCR. mRNA expression level of *ACE2*, *TMPRSS2*, *AGTR1*, *AGTR2*, and *ADAM17* was determined in relation to expression of internal control *GAPDH* mRNA by subtracting Ct of gene of interest (GOI) from *GAPDH*. The bar graphs represent mean ± SE from 6-8 healthy RM.

Supplementary Figure 3 Bar plots of jejunum *ACE2* gene expression are shown in male (n=3) and female (n=3) SIV-uninfected RMs at their pre infection time point. No statistically significant differences of *ACE2* mRNA expression among male and female macaques were detected with paired T-test.

Supplementary Figure 4 Expression of cytokines in the jejunum tissue of uninfected RM as determined by qRT-PCR. mRNA expression of *IL-1β*, *IL-6*, and *TNF-α*. mRNA expression level was determined in relation to expression of internal control *GAPDH* mRNA by subtracting Ct of gene of interest (GOI) from *GAPDH*. The bar graphs represent mean ± SE from 6-8 healthy RM.

Supplementary Figure 5 Bar plots of jejunum ACE2 protein expression are shown in male (n=3) and female (n=3) SIV-uninfected RMs at their pre infection time point. No statistically significant differences of ACE2 protein mean fluorescent intensity (MFI) values among male and female macaques were detected with paired T-test.

Supplementary Figure 6
**(A, B)** Representative immunohistochemistry images of AGTR2 expression detected in a RM (KP54) during pre infection. **(B)** The inset in **(A)** shows AGTR2+ cells in the epithelial region of the jejunum crypts (green arrows). **(C)** Bar plots of jejunum AGTR2+ cells/mm^2^ are shown in male (n=3) and female (n=3) SIV-uninfected RMs at their pre infection time point. No statistically significant differences in the jejunum AGTR2+ cells/mm^2^ among male and female macaques were detected with paired T-test.

Supplementary Figure 7 Bar plots of jejunum TMPRSS2 protein expression in villi epithelium are shown in male (n=3) and female (n=3) SIV-uninfected RMs at their pre infection time point. No statistically significant differences in the area of TMPRSS2+ tissue in villi epithelium among male and female macaques were detected with paired T-test.
