# Supplementary figures and images for: Simian Immunodeficiency Virus Infection Mediated Changes in Jejunum and Peripheral SARS-CoV-2 Receptor ACE2 and Associated Proteins or Genes in Rhesus Macaques
Source: Front Immunol. 2022 Feb 25;13:835686. doi: 10.3389/fimmu.2022.835686 (PMC8914048; doi:10.3389/fimmu.2022.835686)

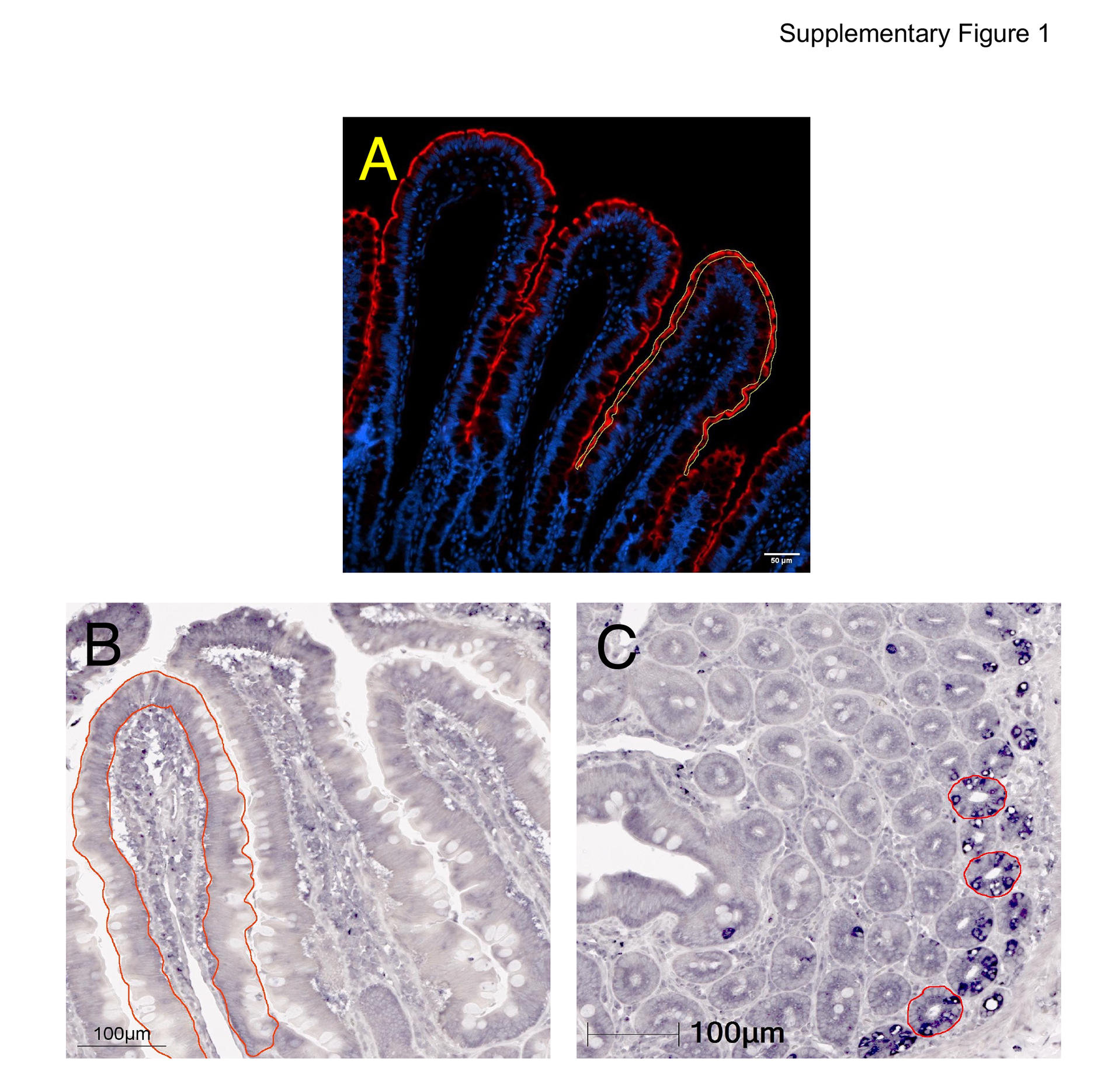

Supplement: Supplementary Figure 1 — The representative figures depict the gating strategy for region of interest (ROI) for ACE2 (A), TMPRSS2 staining in villi (B) and crypts (C). ROIs were manually drawn (in yellow or red lines) in the epithelium of randomly selected villi/crypts for the quantification of ACE2/TMPRSS2 intensity in epithelial cells. [file Image_1.tif]

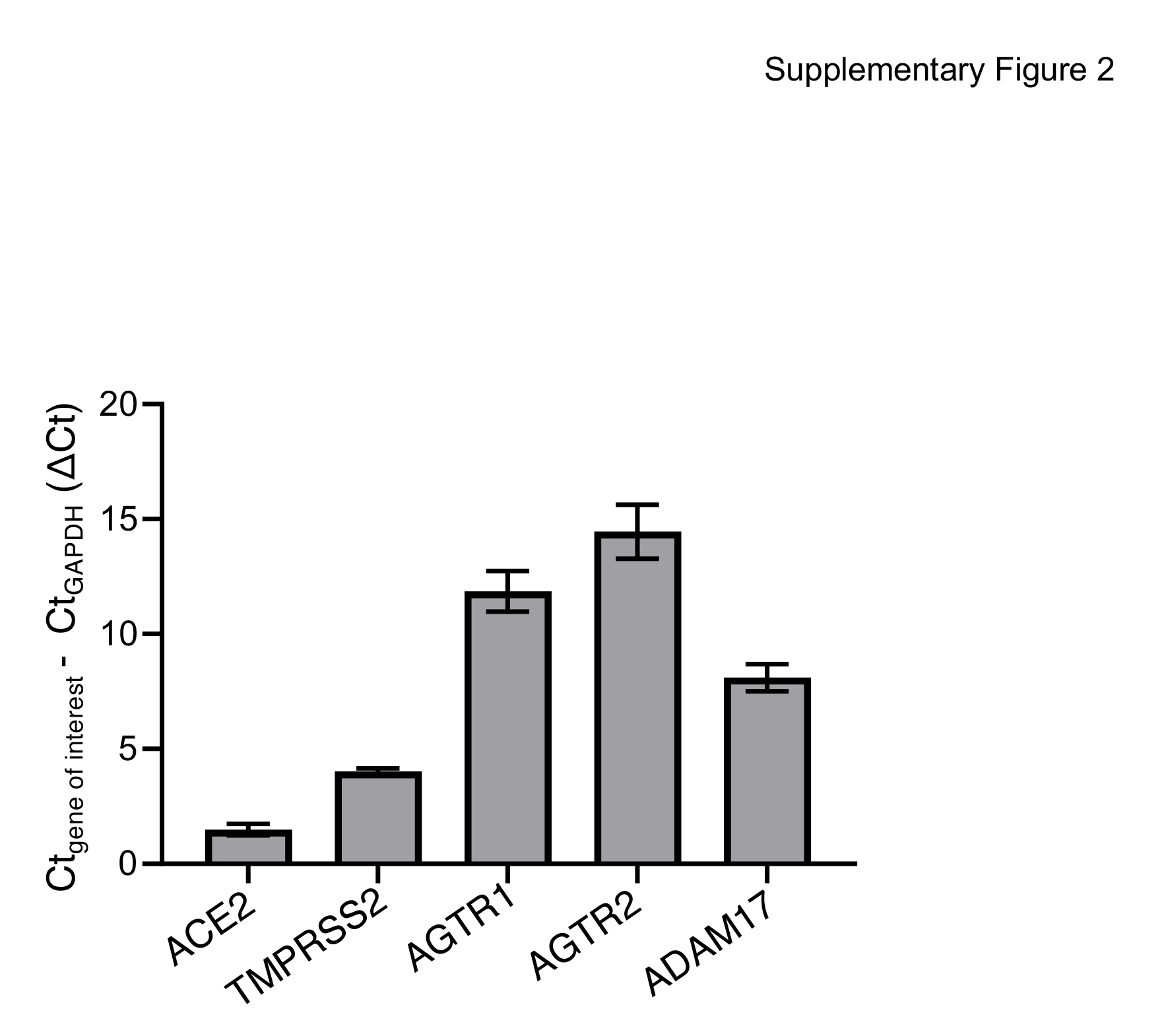

Supplement: Supplementary Figure 2 — Bar plots of ACE2 and associated genes expression in the jejunum tissue of uninfected RM as determined by qRT-PCR. mRNA expression level of ACE2, TMPRSS2, AGTR1, AGTR2, and ADAM17 was determined in relation to expression of internal control GAPDH mRNA by subtracting Ct of gene of interest (GOI) from GAPDH. The bar graphs represent mean ± SE from 6-8 healthy RM. [file Image_2.tif]

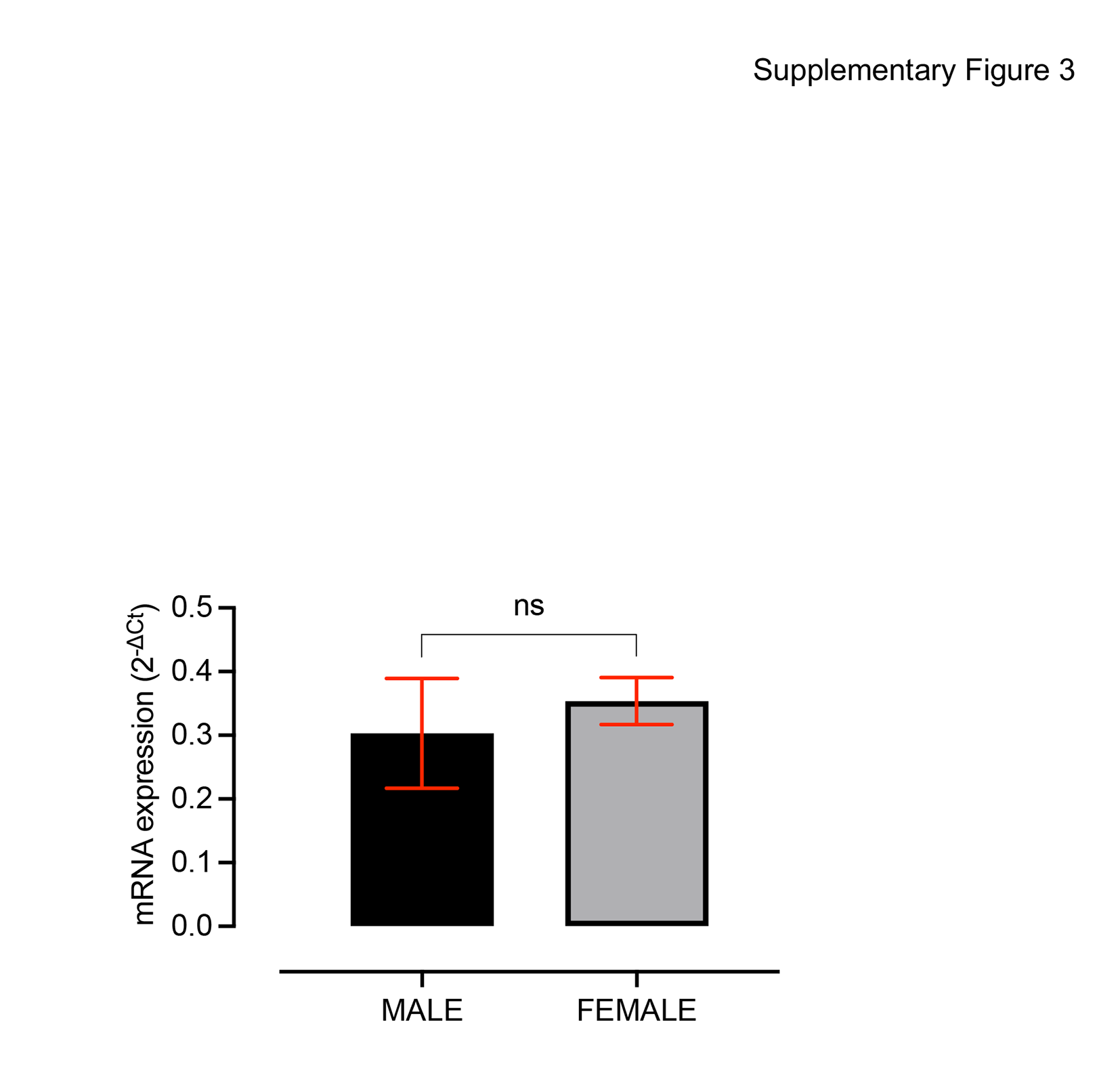

Supplement: Supplementary Figure 3 — Bar plots of jejunum ACE2 gene expression are shown in male (n=3) and female (n=3) SIV-uninfected RMs at their pre infection time point. No statistically significant differences of ACE2 mRNA expression among male and female macaques were detected with paired T-test. [file Image_3.tif]

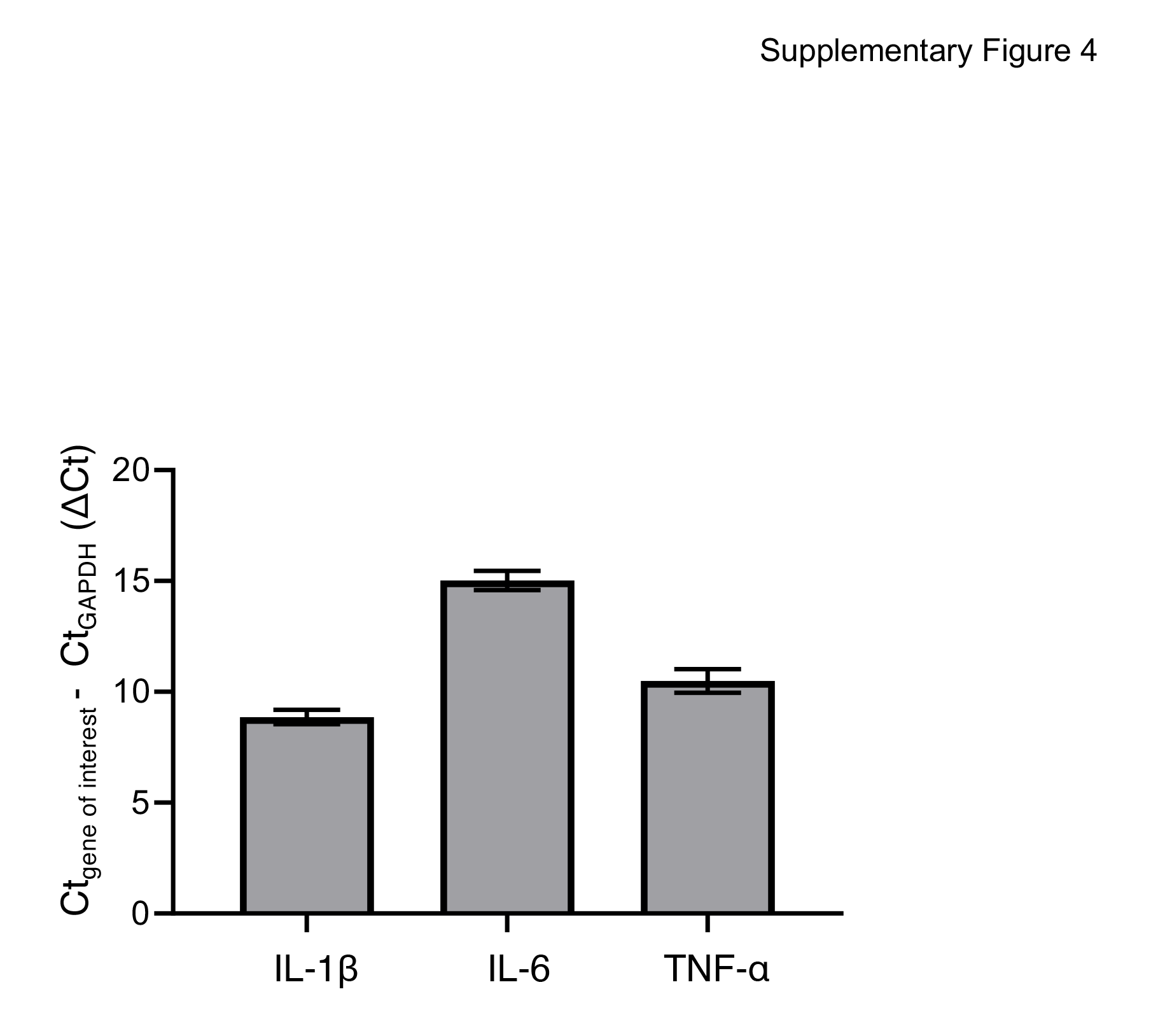

Supplement: Supplementary Figure 4 — Expression of cytokines in the jejunum tissue of uninfected RM as determined by qRT-PCR. mRNA expression of IL-1β, IL-6, and TNF-α. mRNA expression level was determined in relation to expression of internal control GAPDH mRNA by subtracting Ct of gene of interest (GOI) from GAPDH. The bar graphs represent mean ± SE from 6-8 healthy RM. [file Image_4.tif]

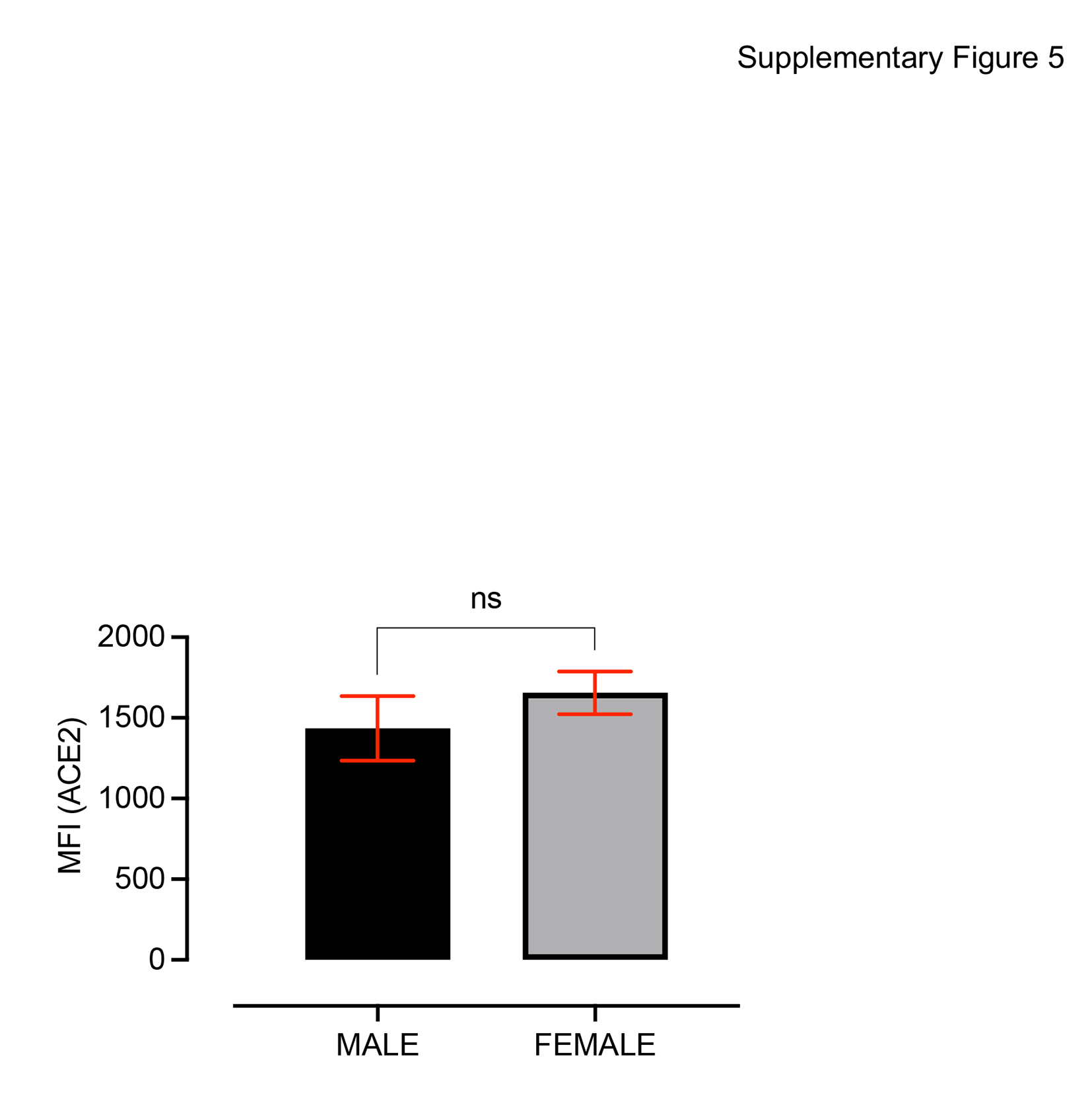

Supplement: Supplementary Figure 5 — Bar plots of jejunum ACE2 protein expression are shown in male (n=3) and female (n=3) SIV-uninfected RMs at their pre infection time point. No statistically significant differences of ACE2 protein mean fluorescent intensity (MFI) values among male and female macaques were detected with paired T-test. [file Image_5.tif]

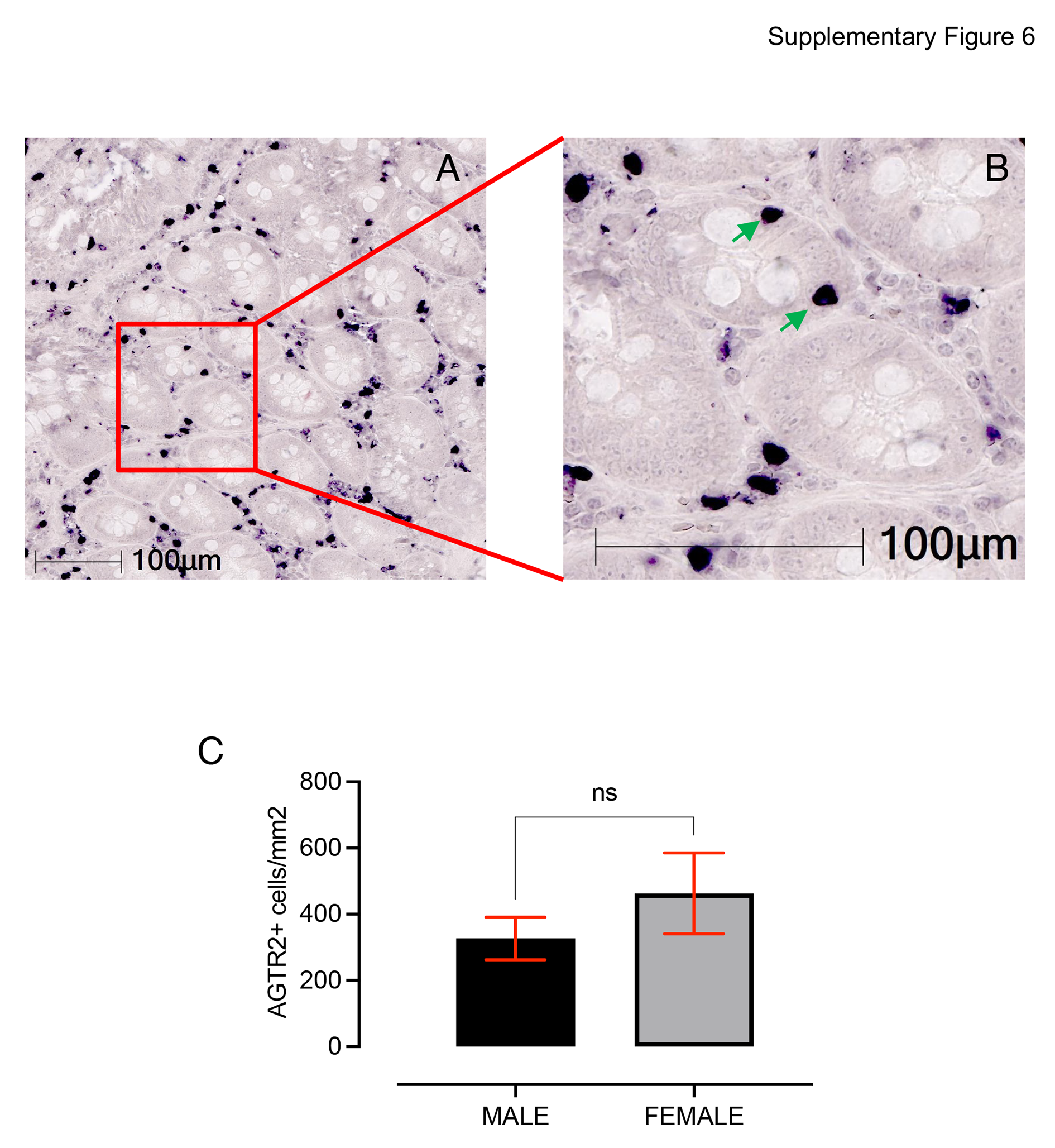

Supplement: Supplementary Figure 6 — (A, B) Representative immunohistochemistry images of AGTR2 expression detected in a RM (KP54) during pre infection. (B) The inset in (A) shows AGTR2+ cells in the epithelial region of the jejunum crypts (green arrows). (C) Bar plots of jejunum AGTR2+ cells/mm2 are shown in male (n=3) and female (n=3) SIV-uninfected RMs at their pre infection time point. No statistically significant differences in the jejunum AGTR2+ cells/mm2 among male and female macaques were detected with paired T-test. [file Image_6.tif]

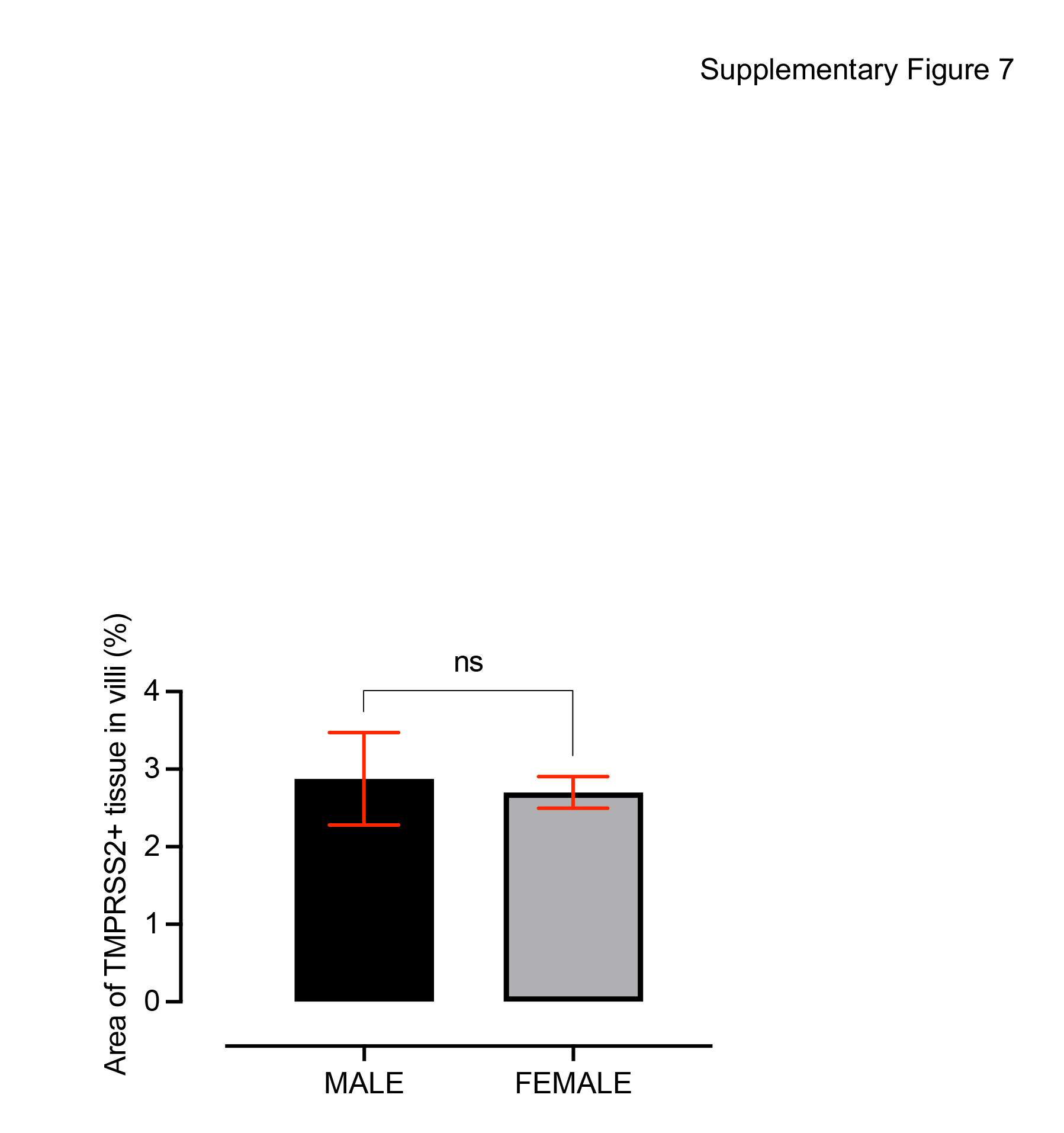

Supplement: Supplementary Figure 7 — Bar plots of jejunum TMPRSS2 protein expression in villi epithelium are shown in male (n=3) and female (n=3) SIV-uninfected RMs at their pre infection time point. No statistically significant differences in the area of TMPRSS2+ tissue in villi epithelium among male and female macaques were detected with paired T-test. [file Image_7.tif]
