# Supplementary material for: Simian Immunodeficiency Virus Infection Mediated Changes in Jejunum and Peripheral SARS-CoV-2 Receptor ACE2 and Associated Proteins or Genes in Rhesus Macaques
Source: Front Immunol. 2022 Feb 25;13:835686. doi: 10.3389/fimmu.2022.835686 (PMC8914048; doi:10.3389/fimmu.2022.835686)
Supplement: Supplementary file 8 [file Table_1.pdf]

Supplementary Table 1. List of antibodies used for identifying proteins of interest in the present study.

Antibody	Isotype	Clone	Assay	Amount (μl) /Dilution	Source
Anti-cow cytokeratin, Wide Spectrum Screening	Rabbit polyclonal IgG	-	IF	1:250	Dako
Anti-human CD3	Mouse IgG1	SP34-2	FC	5	BD Pharmingen
Anti-human CD4	Mouse IgG1	L200	FC	5	BD Pharmingen
Anti-human CD8	Mouse IgG1	SK1	FC	5	Biolegend
Anti-human ACE2	Rabbit polyclonal IgG	-	IF and IHC	1:1000	Sino Biological
Anti-human AGTR2	Rabbit polyclonal IgG	-	IHC	1:100	LS Bio
Anti-human TMPRSS2	Rabbit polyclonal IgG	-	IHC	1:100	Novus Biologicals
Normal Rabbit IgG control	Rabbit IgG	-	IF and IHC	10 μg/mL	R&D Systems

Note: Before all staining, dilution of the respective antibodies was determined after serial dilution experiment.

FC, IF, and IHC denote flow cytometry, Immunofluorescence, and immunohistochemistry, respectively.
