# Supplementary material for: Simian Immunodeficiency Virus Infection Mediated Changes in Jejunum and Peripheral SARS-CoV-2 Receptor ACE2 and Associated Proteins or Genes in Rhesus Macaques
Source: Front Immunol. 2022 Feb 25;13:835686. doi: 10.3389/fimmu.2022.835686 (PMC8914048; doi:10.3389/fimmu.2022.835686)
Supplement: Supplementary file 9 [file Table_2.pdf]

**Supplementary Table 2:** List of primers used in real-time PCR.

| **Target Gene** | **Primer** | **Sequence (5' to 3')** | **Annealing Temp (°C)** | **Product length (bp)** | **Gene ID/ Reference** |
|---|---|---|---|---|---|
| ACE2 | Forward | CAGCCACACCTAAGCATTT | 60 | 150 | NM_001135696.1 |
| | Reverse | TCCACCTCCACTTCTCTAAC | | | |
| ADAM17 | Forward | CGATCCTGGCATCATGTATC | 60 | 131 | XM_028831980.1 |
| | Reverse | TCTTCTGGGCAGTCTCAA | | | |
| AGTR1 | Forward | CCCACTGTTTCCCAGTCATT | 60 | 147 | XM_028843764.1 |
| | Reverse | AGGGTGACACAGGTCATTTC | | | |
| AGTR2 | Forward | TTCCCTTCCATGTTCTGACC | 60 | 191 | Matsushima-Otsuka [1] |
| | Reverse | AAACACACTGCGGAGCTTCT | | | |
| GAPDH | Forward | GAAATCCCATCACCATCTTCCAGG | 60 | 120 | Ahn et al, 2008 [2] |
| | Reverse | GAGCCCCAGCCTTCTCCATG | | | |
| IL-1β | Forward | GGTGTTCTCCATGTCCTTTGTA | 60 | 122 | NM_001042756.1 |
| | Reverse | GTAGCGTGGGCTTATCATCTT | | | |
| IL-6 | Forward | TGGCTGAAAAAGATGGATGCT | 60 | 134 | Abel et al, 2001 [3] |
| | Reverse | TTGCTCCTCACTACTCTCAAACCT | | | |
| TMPRSS2 | Forward | GGATGGTGGCTGGAAATAAA | 60 | 117 | XM_028845315.1 |
| | Reverse | CAAGGGCACTGTCTACATTC | | | |
| TNF-α | Forward | GGCTCAGGCAGTCAGATCATC | 60 | 75 | Abel et al, 2001 [3] |
| | Reverse | GCTTGAGGGTTTGCTACAACATG | | | |

bp: basepairs

[1] Matsushima-Otsuka S, Fujiwara-Tani R, Sasaki T, Ohmori H, Nakashima C, Kishi S, Nishiguchi Y, Fujii K, Luo Y, Kuniyasu H. Significance of intranuclear angiotensin-II type 2 receptor in oral squamous cell carcinoma. Oncotarget. 2018 Nov 27;9(93):36561-36574. doi: 10.18632/oncotarget.26337. PMID: 30564297; PMCID: PMC6290968.

[2] Ahn K, Huh JW, Park SJ, Kim DS, Ha HS, Kim YJ, Lee JR, Chang KT, Kim HS. Selection of internal reference genes for SYBR green qRT-PCR studies of rhesus monkey (Macaca mulatta) tissues. BMC Mol Biol. 2008 Sep 10;9:78. doi: 10.1186/1471-2199-9-78. PMID: 18782457; PMCID: PMC2561044.

[3] Abel K, Alegria-Hartman MJ, Zanotto K, McChesney MB, Marthas ML, Miller CJ. Anatomic site and immune function correlate with relative cytokine mRNA expression levels in lymphoid tissues of normal rhesus macaques. Cytokine. 2001 Dec 7;16(5):191-204. doi: 10.1006/cyto.2001.0961. PMID: 11814315.
