# Supplementary material for: Simian Immunodeficiency Virus Infection Mediated Changes in Jejunum and Peripheral SARS-CoV-2 Receptor ACE2 and Associated Proteins or Genes in Rhesus Macaques
Source: Front Immunol. 2022 Feb 25;13:835686. doi: 10.3389/fimmu.2022.835686 (PMC8914048; doi:10.3389/fimmu.2022.835686)
Supplement: Supplementary file 10 [file Table_3.pdf]

Supplementary Table 3: 26 significant upstream regulators for gene ACE2 based on all species

Upstream Regulator	Molecule Type	Predicted Activation State	Activation z-score	p-value of overlap	Target Molecules in Dataset
lipopolysaccharide	chemical drug		0.352	8.31E-08	ABCC2,ABCC5,ABCF2,ABCG8,**ACE2**,ADORA1,AFF3,APOA4,APOB,AQP3,C5AR1,CCN4,CCR6,CD4,CD44,CD74,CDH5,COL12A1,COL1A1,COL6A1,CYBA,DOCK8,FABP2,FASN,FN1,GUCY1B1,HPGD,HSPA13,IL7R,KIT,LIF,LMNB1,MARCKSL1,MARCO,MEF2C,MME,MMP7,MTSS1,MTTP,MYBL2,MYC,MYO9A,NOS1,NOTCH1,NR1H4,OPRM1,PCK2,PDIA6,PFKFB3,PHLDA1,PHLDB2,PIK3CG,PIM1,PLAT,PTAFR,RAB37,RHBDF2,SEC24D,SELE,SELENOP,SLC12A2,SLC28A2,SLC30A1,SLC5A1,STAT1,STXBP1,SYNPO,TAGAP,TAP1,TNFSF4,TP53,TP63,UCP2,UGT1A1,XDH
HNF1A	transcription regulator	Inhibited	-4.247	0.000000429	ABCC2,**ACE2**,ALDOB,**ANPEP**,APOB,APOC3,AQP3,BMP3,CDHR2,CLIC5,**DPP4**,**ENPEP**,FBP1,GPX2,HNF4G,MTTP,NR1H4,PDHX,RAB37,RACK1,SI,SLC10A2,SLC5A1,UCP2,UGT1A1,UGT2B15,VIL1
AGT	growth factor		0.321	0.00000048	**ACE2**,ADAMTS2,BMP1,C5AR1,CCN4,CD44,CDH5,CGREF1,COL12A1,COL15A1,COL1A1,COL6A1,COL6A2,COL6A3,CYBA,CYP2J2,ENPEP,ETV4,FASN,FN1,FRZB,HIP1,LIF,LRP8,LRRC7,MYC,MYH11,NDRG1,NOS1,NOX5,PARP1,PREX1,PRSS2,RUNX1,SCN2A,SELE,SFRP4,SLC5A1,SLC6A2,SOX4,TP53,UCP2
SMARCA4	transcription regulator		-1.406	0.00000094	**ACE2**,AGR2,CCR6,CD44,CD74,COL1A1,COTL1,CUL4B,CYP26A1,DYSF,FABP6,FBP1,FN1,GSTO1,HPGD,IL7R,ITPR1,KCNS3,KIT,LMNA,MEF2C,MMP7,MUC1,MYC,MYH11,PHLDB1,PLAT,PLS1,SELENOP,TAP1,TP53,TUBB,UBD
GATA4	transcription regulator		-1.071	0.00000899	ABCG8,**ACE2**,ADORA1,COL1A1,FABP6,FRZB,FSTL4,KIT,MEF2C,MYC,PYY,RYR2,SCN5A,SFRP4,SI,SLC10A2,SOX9
LEPR	transmembrane receptor		-0.622	0.00000929	ABCG8,**ACE2**,ACOX1,APOA4,APOB,COL1A1,FASN,FN1,LGMN,LIF,MMP7,MTTP,NOX3,SELE,UCP2
androgen	chemical drug		-1.633	0.0000141	**ACE2**,AGR2,CD44,COL12A1,EZH1,FLNA,GGT1,MYC,PIM1,RUNX1,SOX4,SOX9,UGT2B15,VIL1
atorvastatin	chemical drug		0.515	0.0000186	ABCC2,ABCG8,**ACE2**,AGR2,APOB,APOC3,COL1A1,CYBA,MTTP,MVD,NOS1,PLAT,SELE,SLC10A2,TP53
D-glucose	chemical - endogenous ma		-0.813	0.0000293	**ACE2**,ACOX1,ALDOA,ALDOB,ALDOC,APOB,APOC3,COL1A1,COL2A1,CYBA,FABP2,FASN,FBP1,FN1,GIPR,ITPR1,LCP1,MYC,MYH11,NR1H4,PARP1,PCK2,PDHX,PDIA6,SELE,SELENOP,SLC5A1,SOX9,STXBP1,TP53,UCP2,VIL1
MYOCD	transcription regulator		-0.128	0.000113	**ACE2**,COL1A1,COL2A1,FN1,HCN4,MEF2C,MYH11,SCN5A,TP53
IFNG	cytokine		-1.469	0.000175	**ACE2**,ADORA1,AGPAT1,C5AR1,CCL23,CCR6,CD4,CD44,CD74,CDH5,CLIC5,COL1A1,CYBA,DAPK1,DPP4,FASN,FBP1,FN1,GPER1,H2BC18,HIP1,IL7R,ITPR1,LIF,MARCKSL1,MTSS1,MUC1,MYC,NOS1,NOTCH1,NOX3,PFKFB3,PHLDA1,PIM1,PRKG2,PTAFR,RACK1,RFXANK,SELE,SELENOP,SFRP1,SLC12A2,SLC28A2,SMC1B,STAT1,TAP1,TP53,TP63,UBD
Esrra	transcription regulator		-0.971	0.000186	**ACE2**,ACOX1,ALDOA,ALDOB,ALDOC,APOA4,FABP2,FBP1,PCK2,SOX9
enalapril	biologic drug		0.933	0.00068	ABCC2,**ACE2**,COL1A1,CYBA,FN1,GPX2,MYC,UCP2
pioglitazone	chemical drug		-0.128	0.00439	**ACE2**,APOA4,APOB,APOC3,FBP1,FN1,HPGD,MUC1,UCP2
glucocorticoid	chemical drug		0.817	0.00454	ABCC8,**ACE2**,CD44,CD74,COL1A1,CRIP1,FASN,FN1,MYC,NOS1,PLAT,SELE,TP53
NFE2L2	transcription regulator		-0.95	0.00591	ABCC2,ABCG2,**ACE2**,ALDOA,AOX1,APOA4,COL1A1,FN1,GPX2,GSTO1,LMNA,MEF2C,PDIA6,RACK1,SEC23A,SLC38A3,TP53,UGT1A1
TBX5	transcription regulator		-1.109	0.00766	**ACE2**,COL1A1,MEF2C,RYR2,SCN5A,TNFSF4
IFN Beta	group		-1.223	0.00907	**ACE2**,CD74,DPP4,ENPEP,FN1,KIT,MYC,STAT1,TP53
AGTR1	G-protein coupled recepto		0.277	0.0132	**ACE2**,CYBA,FN1,XDH
HEXIM1	transcription regulator			0.0191	**ACE2**,COL1A1,TP53
MEF2C	transcription regulator		-1.109	0.0262	**ACE2**,COL1A1,COL2A1,FRZB,MEF2C,RYR2,SCN5A
miR-4658 (and other miRNAs w/seed UGAGUGU)	mature microRNA	Activated	2.111	0.0278	**ACE2**,C1orf115,COL15A1,DPP4,FCRL1,MEP1A,NUDT12,RASL12,RFXANK,RPS17,SNAP91
ramipril	chemical drug			0.0279	**ACE2**,COL1A1,FN1
KCNE3	ion channel			0.028	**ACE2**,CDHR5,PHLDA1,SLC2A5
miR-4760-5p (and other miRNAs w/seed UUAGAUU)	mature microRNA	Activated	2.887	0.0382	ABCG2,**ACE2**,ALDOB,ASIC1,COL15A1,CYYR1,DENND4A,ENPEP,FAM13C,MRPS2,SIRPG,TNFSF4
miR-136-3p (miRNAs w/seed AUCAUCG)	mature microRNA	Activated	2.887	0.0426	**ACE2**,AFF3,C2CD2L,CBLN2,FABP2,FAM13C,KLK6,PDHX,PHLDA1,SEC23A,SEC24D,VIL1
